# Divergent Roles of SmHMGR2 and a Novel SmHMGR5 in Tanshinone Biosynthesis Revealed by CRISPR/Cas9-Mediated Knockout in *Salvia miltiorrhiza*

**DOI:** 10.3390/ijms27083485

**Published:** 2026-04-13

**Authors:** Ziting Lan, Mei Tian, Jianing Liu, Wenlong Shi, Tong Chen, Qing Ma, Baolong Jin, Yujun Zhao, Haiyan Zhang, Chang-Jiang-Sheng Lai, Guanghong Cui

**Affiliations:** 1 State Key Laboratory for Quality Ensurance and Sustainable Use of Dao-di Herbs, National Resource Center for Chinese Materia Medica, China Academy of Chinese Medical Sciences, Beijing 100700, China; 2 Dalian Institute of Marine Traditional Chinese Medicine, China Academy of Chinese Medical Sciences, Dalian University, Dalian 116622, China

**Keywords:** *Salvia miltiorrhiza*, HMGR, CRISPR/Cas9, tanshinone biosynthesis, functional divergence

## Abstract

3-Hydroxy-3-methylglutaryl coenzyme A reductase (HMGR) serves as a key rate-limiting enzyme in the mevalonate pathway and plays a central regulatory role in the biosynthesis of tanshinones. To date, four HMGR family members (*SmHMGR1–4*) have been identified in *Salvia miltiorrhiza*. Here, we cloned and identified a novel member, *SmHMGR5*, by integrating multiple genomic datasets. Genomically, *SmHMGR5* formed an inverted repeat with *SmHMGR3* (98.04% homology) and phylogenetically clustered with SmHMGR2. Based on the expression patterns of the five HMGR genes, we further generated *SmHMGR2* and *SmHMGR5* knockout mutants using CRISPR/Cas9 technology and compared their effects on the accumulation of 12 tanshinones and 4 phenolic acids via UPLC-MS-based metabolomic analysis. Knockout of *SmHMGR2* significantly suppressed the accumulation of seven tanshinones, whereas *SmHMGR5* knockout downregulated only three tanshinones, and neither mutation affected phenolic acids. Notably, the major compound tanshinone IIA remained stable across different mutants, but tanshinone IIB was markedly reduced upon *SmHMGR2* knockout, suggesting complex regulatory mechanisms in tanshinone biosynthesis. These findings provide new insights into the biosynthetic network of tanshinones and establish a theoretical foundation for metabolic engineering strategies aimed at enhancing the production of bioactive constituents in *S. miltiorrhiza*.

## 1. Introduction

*Salvia miltiorrhiza* Bunge is a perennial medicinal plant of the genus *Salvia* in the family *Lamiaceae*. As a core medicinal herb in traditional Chinese medicine with the effects of “activating blood circulation to remove blood stasis and dredging meridians to alleviate pain” [1], it also serves as a model species for medicinal plant research [2,3,4]. Its main active components consist of lipophilic tanshinones (e.g., cryptotanshinone, tanshinone IIA) and water-soluble salvianolic acids (e.g., rosmarinic acid, salvianolic acid B). The former exerts anti-platelet aggregation and vasodilatory effects [5,6,7], while the latter exhibits significant anti-inflammatory and antioxidant activities [8,9,10]. The content and ratio of these two types of components directly affect the medicinal quality of *S. miltiorrhiza*. Therefore, elucidating the biosynthetic regulatory mechanisms of these two classes of compounds is of great significance for improving the quality and medicinal value of *S. miltiorrhiza*.

In *S. miltiorrhiza*, the biosynthesis of tanshinones mainly relies on two core metabolic pathways: the mevalonate (MVA) pathway and the 2-methyl-D-erythritol 4-phosphate (MEP) pathway. In the MVA pathway, 3-hydroxy-3-methylglutaryl coenzyme A reductase (HMGR) is a key rate-limiting enzyme that catalyzes the irreversible conversion of 3-hydroxy-3-methylglutaryl coenzyme A (HMG-CoA) to mevalonate (MVA), thereby directly regulating the supply of downstream isopentenyl pyrophosphate (IPP) [11]. IPP produced by both the MVA and MEP pathways serves as the common precursor for tanshinones. It is first converted into geranylgeranyl pyrophosphate (GGPP), which is then modified by downstream diterpene synthases (diTPSs), cytochromes P450 (CYPs), and 2-oxoglutarate-dependent dioxygenases (2OGD) to form various tanshinones [12,13,14,15,16,17].

To date, four HMGR family members (*SmHMGR1-4*) have been identified in *S. miltiorrhiza.* Phylogenetic analysis shows that this family can be divided into two subclusters: *SmHMGR1* (GenBank accession No. EU680958.1) and *SmHMGR4* (JN831103.1) cluster into one clade, while *SmHMGR2* (FJ747636.1) and *SmHMGR3* (JN831102.1) belong to the other, implying potential functional redundancy or divergence within each clade [18]. *SmHMGR1* is the first cloned and characterized member of this family [19]. Either overexpressing *SmHMGR1* alone, or co-expressing it with key terpenoid biosynthetic genes such as *SmGGPPS* and *SmDXR*, can effectively increase the accumulation of tanshinones [20,21]. Overexpression of *SmHMGR4* also promotes tanshinone accumulation in *S. miltiorrhiza* roots [22]. As for *SmHMGR2* and *SmHMGR3*, overexpression of *SmHMGR2* significantly promotes the accumulation of dihydrotanshinone, cryptotanshinone, and tanshinone I [23]. By editing the TTEGCLVA domain (TTGGTC → AAGAGA) of SmHMGR3 using the Prime editing system, the contents of tanshinone I, dihydrotanshinone, and salvianolic acid B were significantly increased in some mutant lines [24]. Thus, apart from *SmHMGR3*, the functions of the other three HMGR genes have been primarily investigated through overexpression-based studies, with a lack of mutant data. The establishment of an efficient CRISPR/Cas9 system in *S. miltiorrhiza* inbred line bh2-7 now provides a foundation for studying the precise functions of individual genes in tanshinone and salvianolic acid compounds biosynthesis [25].

Here, based on the transcriptome data derived from the *S. miltiorrhiza* bh2-7 genome, and through sequence alignment, syntenic analysis, and phylogenetic analysis, we identified and cloned a novel family member, designated SmHMGR5. Using transcriptome data from the IMP database, we further analyzed the organ-specific expression patterns of the HMGR family, which comprises five homologous genes (*SmHMGR*1–5). Based on their expression patterns across different organs and phylogenetic relationships, we selected *SmHMGR2* as a representative family member. It is highly expressed in most organs and clusters together with SmHMGR5 in the same clade. To address the gaps in functional characterization of the HMGR family and overcome the limitations of previous studies that relied primarily on overexpression, we employed an efficient CRISPR/Cas9 system established in *S. miltiorrhiza* to generate targeted knockout mutants of *SmHMGR5* and *SmHMGR2*, the latter of which lacks detailed loss-of-function data. Metabolomic analysis of the resulting homozygous and biallelic mutants revealed distinct regulatory mechanisms of these two genes in tanshinone compound biosynthesis, highlighting their functional diversification. Collectively, this study not only clarifies the precise roles of specific *HMGR* family members in modulating the accumulation of bioactive compounds but also establishes a foundation for precision metabolic engineering aimed at improving the medicinal quality of *S. miltiorrhiza*.

## 2. Results

### 2.1. Identification and Sequence Analysis of SmHMGR5 in S. miltiorrhiza

**Figure 1:**
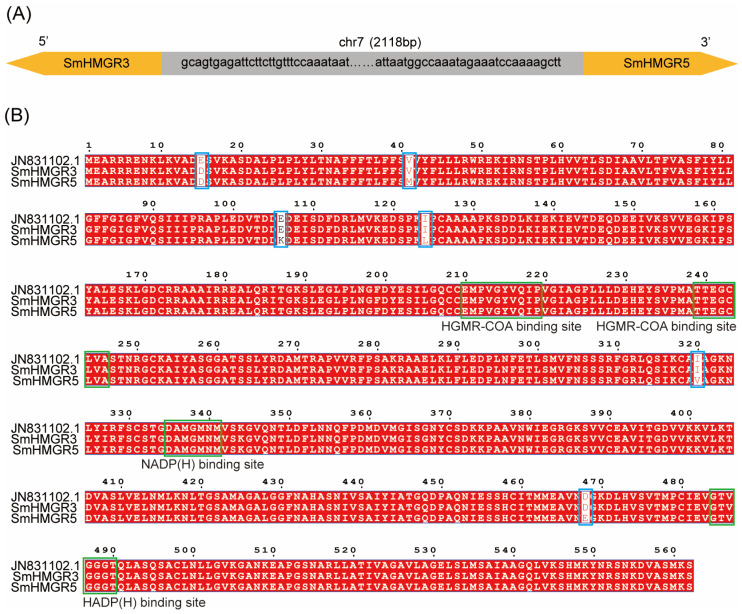
Chromosomal Location and sequence alignment of SmHMGR3 and SmHMGR5. (**A**) Schematic representation of tandem arrangement of *SmHMGR3* and *SmHMGR5* on *S. miltiorrhiza* chromosome 7. (**B**) Multiple sequence alignment of amino acids among SmHMGR3, SmHMGR5, and the reference sequence JN831102.1. Red backgrounds indicate amino acid residues that are completely conserved across all three sequences. Blue boxes highlight the 6 amino acid substitution sites distinguishing SmHMGR5 from SmHMGR3 (positions 15, 41, 106, 124, 320, and 470). Green boxes mark the two functional HMG-CoA binding motifs (EMPVGYVQIP and TTEGCLVA) and two NADP(H) binding motifs (DAMGMNM and GTVGGGT) in the protein.

Five annotated genomic datasets of *S. miltiorrhiza* have been publicly released, among which the genomes of DSS3, bh2-7, and shh have been assembled to the scaffold or chromosomal level (Supplementary Table S1). We selected these three genomes to compare their annotation of *SmHMGRs*. In shh, four *SmHMGRs* were annotated, but there was no annotated homolog of *SmHMGR2* (FJ747636.1), while *SmHMGR3* had two annotations with 97.99% similarity [26]. In the genomes of DSS3 and bh2-7, in addition to *SmHMGR1*, *SmHMGR2,* and *SmHMGR4*, there are also two homologous genes with high sequence similarity to *SmHMGR3* [16,27]. In the bh2-7 genome, these two genes are located in adjacent regions with opposite transcription directions, which are designated as IMPTSMI1N39000 and IMPTSMI1N39001 in the Integrated Medicinal Plantomics database (https://www.bic.ac.cn/IMP/; accessed on 4 March 2025), with a sequence similarity of 98.04% (Figure 1A). In DSS3, the corresponding two genes (GWHTAOSJ026839 and GWHTAOSJ026840) have a similarity of 98.32% and are also tandemly repeated with opposite transcriptional orientations (Supplementary Table S1).

Subsequently, we performed a multiple sequence alignment between the two highly homologous genes in bh2-7 and *SmHMGR3* (JN831102.1) [18]. The results show that the amino acid sequence of IMPTSMI1N39001 differs from SmHMGR3 by only a single amino acid substitution (E → D) at position 15. IMPTSMI1N39000 differed from SmHMGR3 by six amino acids, located at positions 15 (E → D), 41 (M → V), 106 (K → E), 124 (L → I), 320 (V → I), and 470 (E → D) (Figure 1B). Therefore, IMPTSMI1N39001 in bh2-7 corresponds to *SmHMGR3*, while IMPTSMI1N39000 is a novel member of the *HMGR* family.

To verify the identity of IMPTSMI1N39000, we designed specific primers based on the sequence prediction results of the bh2-7 genome and successfully cloned the complete coding region sequences of IMPTSMI1N39000 and *SmHMGR3* from bh2-7 inbred line. The experimentally determined sequences of the two genes are identical to the genome-predicted ones, with a full length of 1689 bp for both, a theoretical isoelectric point of 4.90 for both, and theoretical molecular weights of 139.91 kDa and 139.98 kDa, respectively. Transmembrane structure predictions showed that neither protein contains a transmembrane helical region, and both genes contain only a single exon and no introns. Both genes contain two HMG-CoA binding motifs (“EMPVGYVQIP” and “TTEGCLVA”) and two NADP(H)-binding motifs (“DAMGMNM” and “GTVGGGT”), which are consistent with the conserved motifs reported in the previously identified four *SmHMGRs* (Figure 1) [28,29,30]. Therefore, IMPTSMI1N39000 is designated as *SmHMGR5*.

### 2.2. Evolution and Lineage-Specific Expansion of HMGR Genes in the Lamiaceae Family

Previous phylogenetic analysis has shown that HMGR in dicotyledonous plants can be divided into two independent evolutionary clades, which may originate from an ancient gene duplication event before the differentiation of core eudicots. Most dicotyledonous plants (e.g., *Arabidopsis thaliana*, *Citrus sinensis*) usually contain two HMGR paralogs [31]. However, lineage-specific expansion caused by gene duplication events has led to a significant increase in the copy number of HMGR genes in some species. For example, the number of HMGR family members ranges from 3 to 9 in *Carica papaya*, *Populus trichocarpa*, *Gossypium raimondii*, *Gossypium arboreum* and *Gossypium hirsutum* [31,32,33].

**Figure 2:**
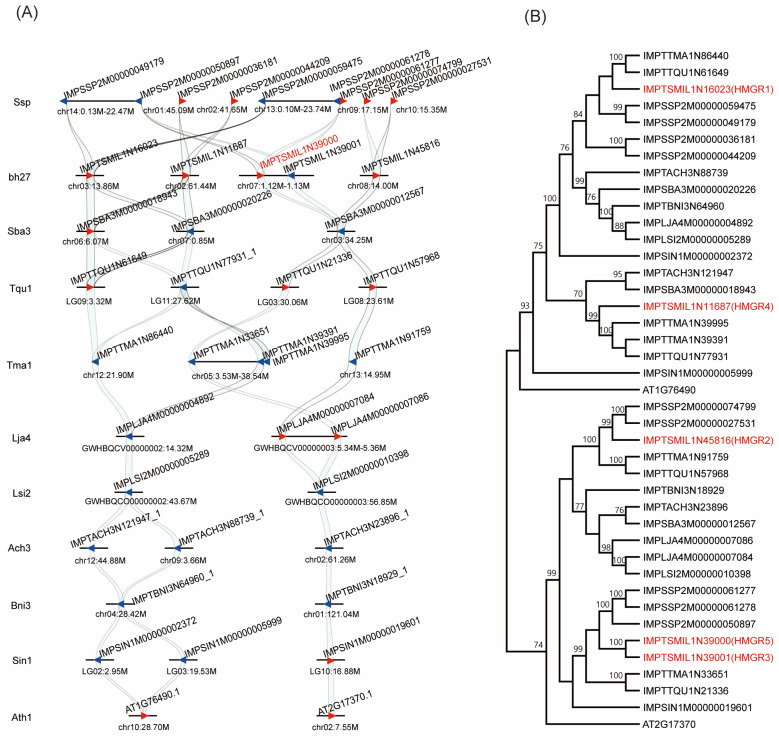
Syntenic relationships and phylogenetic classification of the HMGR gene family in *S. miltiorrhiza* and representative *Lamiaceae* species. (**A**) Syntenic analysis of HMGR genes in *S. miltiorrhiza* bh2-7 and other eight *Lamiaceae* species, together with *Sesamum indicum* from Pedaliaceae and the model species *Arabidopsis thaniana* as out groups, which showing *SmHMGR3* and *SmHMGR5* were inverted repeat sequences in the genome (IMPTSMI1N39000 and IMPTSMI1N39001). Ssp: *Salvia splendens*, bh27: *S. miltiorrhiza*, Sba: *Scutellaria baicalensis*, Tqu: *Thymus quinquecostatus*, Tma: *Thymus mandschuricus*, Lja: *Leonurus japonicus*, Lsi: *Leonurus sibiricus*, Ach: *Ajuga chamaepitys*, Bni: *Ballota nigra*, Sin: *Sesamum indicum*, Ath: *Arabidopsis thaniana.* The blue and red arrows represent different transcription directions. (**B**) Maximum-likelihood phylogenetic tree of HMGR proteins from 11 representative species. The five SmHMGRs are highlighted in red, with all sequences divided into two major evolutionary groups.

To elucidate the evolution of HMGR family genes in *Lamiaceae*, to which *S. miltiorrhiza* belongs, we performed a synteny analysis of 9 representative *Lamiaceae* species using GenoCGLLM [34]. The model plant *Arabidopsis thaliana* was used as an outgroup, and the sesame (*Sesamum indicum*) from Pedaliaceae, a family closely related to *Lamiaceae*, was also included in the analysis. The results showed that HMGR family genes exhibited a dynamic evolutionary pattern. Consistent with the model dicot *Arabidopsis thaliana*, some *Lamiaceae* species retained two HMGR genes; however, other species underwent obvious species-specific expansion. Specifically, we identified 3 HMGR genes in *Scutellaria baicalensis*, 5 in *S. miltiorrhiza*, and up to 9 in *Salvia splendens* (Figure 2A). These differences indicate that tandem duplication and subsequent species-specific gene family expansion are the main driving forces for the evolution of the HMGR gene family in the *Lamiaceae* lineage.

To further clarify the evolutionary relationships of these genes, we constructed a phylogenetic tree using the protein sequences encoded by the above genes. The results showed that they could be clearly divided into two evolutionary clades. SmHMGR1 (IMPTSMI1N16023) and SmHMGR4 (IMPTSMI1N11687) clustered into clade I, which was consistent with the previous report [18]. While SmHMGR2 (IMPTSMI1N45816), SmHMGR3 and SmHMGR5 clustered into clade II (Figure 2B). In *Arabidopsis thaliana*, AtHMGR1 (AT1G76490) and AtHMGR2 (AT2G17370) exhibit significant differentiation in expression patterns and play distinct roles in basal metabolism versus specific developmental or stress responses [35]. Therefore, *SmHMGRs* may also undergo similar functional differentiation, and their specific roles in secondary metabolic regulation warrant further investigation.

### 2.3. Expression Profiles of SmHMGR Genes Revealed by Integrated Analysis of IMP Database Resources

**Figure 3:**
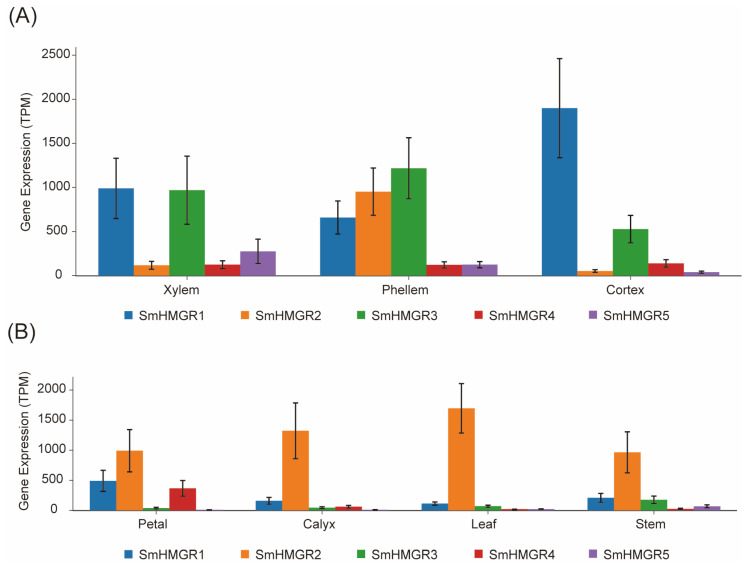
Expression profiles of the five SmHMGRs in bh2-7. The data were obtained from the IPM database. (**A**) Transcription levels of SmHMGRs in the cortex, phellem, and xylem of the roots from the bh2-7. (**B**) Transcription levels of SmHMGRs in the petals, calyxes, leaves, and stems from the bh2-7.

The Integrated Medicinal Plantomics Database (https://www.bic.ac.cn/IMP/; accessed on 26 April 2025) enabled us to investigate gene expression levels across various organs and treatment conditions [36]. This database integrates eight transcriptome datasets, covering different organs, hairy root samples at various time points after methyl jasmonate treatment, and knockout mutants carrying mutations in key enzyme genes of the tanshinone biosynthesis pathway. Among them, four datasets are derived from the bh2-7 line, while the remaining four come from unknown lines. After precise alignment of the five genes’ expression levels across the different datasets were extracted (Supplementary Figure S1). It showed the five SmHMGRs exhibited significantly different expression patterns across organs; the expression levels of SmHMGR1, SmHMGR2 and SmHMGR3 were relatively high across different datasets. Specifically, as observed in the root of bh2-7 (Figure 3), SmHMGR1 and SmHMGR3 were highly expressed in the phellem, cortex, and xylem, while SmHMGR2 was specifically expressed in the phellem, which is considered the main site for the biosynthesis and accumulation of tanshinones [13]. SmHMGR2 also had the highest expression in the upper organs like the leaf, stem, petal, and calyx of bh2-7. In contrast, SmHMGR4 and SmHMGR5 had relatively low expression levels in all organs.

Based on the above expression characteristics, we selected *SmHMGR2* as the representative HMGR gene. It is highly expressed in all organs and belongs to Clade II, in which the newly identified *SmHMGR5* is also included. Thus, both *SmHMGR2* and the newly cloned *SmHMGR5* were selected for gene knockout and functional verification in subsequent studies.

### 2.4. Construction and Analysis of SmHMGR2 and SmHMGR5 Homozygous Mutants Mediated by CRISPR/Cas9

**Figure 4:**
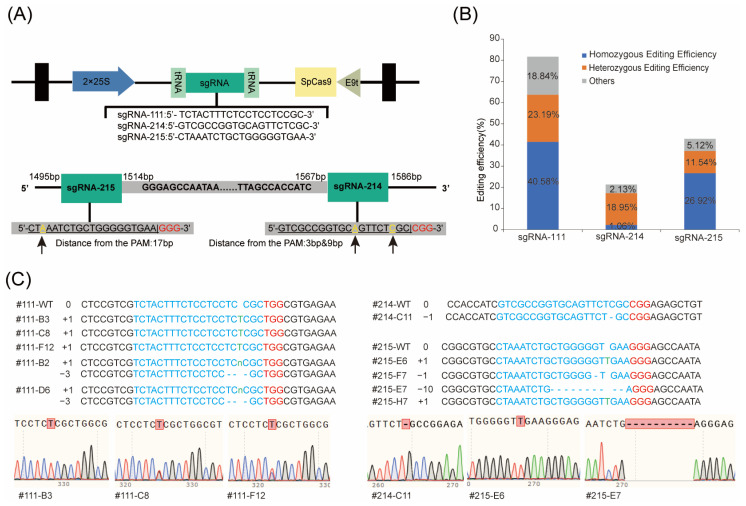
CRISPR/Cas9-Mediated HMGRs Gene Editing. (**A**) The above figure illustrates the schematic representation of the partial pZKD672 plasmid, including Cas9 and gRNA expression cassettes [37]. The figure below shows the relative positions of sgRNA-214 and sgRNA-215 in *SmHMGR5*.The bases marked in yellow are the variant sites compared with the *SmHMGR3* sequence, and in red are the PAM sites. (**B**) The editing efficiency of three sgRNAs. (**C**) Homozygous or biallelic mutants used for metabolomics analysis. The left panel shows the specific edited sequences and insertion/deletion (indel) events (e.g., +1, −1, −3) of each mutant line, in which the PAM sequence “GGG” is highlighted in red, the target sequence is shown in blue, and th inserted bases are marked in green; the right panel presents the corresponding Sanger sequencing chromatograms of the mutants.

In this study, the pZKD672 vector was used as the backbone to construct gene editing vectors [37], and 1–2 target sites were designed for *SmHMGR2* and *SmHMGR5*, respectively. For *SmHMGR2*, a target guide sequence sgRNA-111 (TCTACTTTCTCCTCCTCCGC) was designed based on the *SmHMGR2* gene sequence, corresponding to the sequence position of 107–126 bp. For *SmHMGR5*, two editing sites were designed in the regions of 1495–1514 bp and 1567–1586 bp, namely sgRNA-215 (TTCACCCCCAGCAGATTTAG) and sgRNA-214 (GTCGCCGGTGCAGTTCTCGC) (Figure 4A).

Through the *Agrobacterium rhizogenes* C58C1-mediated genetic transformation system, the above three recombinant vectors (sgRNA-111, sgRNA-214 and sgRNA-215) were used for the infection and induction of hairy roots, and 69, 94 and 78 hairy root lines were obtained, respectively. The transformed hairy root with the empty vector pZKD672 was used as the wild-type control. The genomic DNA of these hairy roots was extracted, and specific primers for *SmHMGR2* and *SmHMGR5* were designed for Sanger sequencing. The sequencing results were analyzed using Synthego ICE software (https://ice.synthego.com), and finally 57, 20 and 34 successfully edited mutants were obtained, respectively. The sequencing results showed that among all sgRNA-mediated gene editing events, the knockout efficiency of *SmHMGR2* mediated by sgRNA-111 was the highest (82.61%), including 40.58% homozygous/biallelic mutations and 23.19% heterozygous/chimeric mutations. Followed by the knockout of *SmHMGR5* mediated by sgRNA-215, with an editing efficiency of 43.58%, the proportions of homozygous/biallelic mutations and heterozygous/chimeric mutations were 26.92% and 11.54%, respectively. The knockout efficiency of *SmHMGR5* mediated by sgRNA-214 was the lowest (21.28%), with only 1.06% homozygous/biallelic mutations and 18.09% heterozygous/chimeric mutations (Figure 4B,C).

Among 57 *SmHMGR2* mutants mediated by sgRNA-111, three homozygous single-base insertion mutant lines (111-B3, 111-C8, 111-F12) and two biallelic mutant lines carrying both a single-base insertion and a 3 bp deletion (111-B2 and 111-D6) were selected for further analysis. From 54 *SmHMGR5* mutants, one single-base deletion mutant line mediated by sgRNA-214 (214-C11) and four mutant lines mediated by sgRNA-215 (215-E6, 215-E7, 215-F7, 215-H7) were selected. Among them, 215-E6 and 215-H7 were single-base insertion mutants, 215-E7 was a 10 bp deletion mutant, and 215-F7 was a single-base deletion mutant (Figure 4C).

Due to the high sequence homology between *SmHMGR3* and *SmHMGR5* (Figure 4A), we examined the sgRNA-214 and sgRNA-215-mediated mutant lines for potential off-target effects. The above selected five homozygous mutants (214-C11, 215-E6/E7/F7/H7) were selected for PCR amplification and verified by Sanger sequencing with *SmHMGR3*-specific primers. The results confirmed that *SmHMGR3* remained unedited in these mutants, indicating that sgRNA-214 and sgRNA-215 could specifically target *SmHMGR5* and no off-target editing occurred at its homologous gene *SmHMGR3* (Supplementary Figure S2). In total, 5 *SmHMGR2*-knockout (*SmHMGR2*-KO) and 5 *SmHMGR5*-knockout (*SmHMGR5*-KO) lines were selected for further analysis.

### 2.5. Expression Analysis of Homologous Genes After Targeted Knockout of SmHMGR2 and SmHMGR5

To determine whether the transcriptional level of the target genes was altered in the aforementioned knockout mutants, we selected typical mutants for validation by quantitative real-time PCR. The expression level of the five *SmHMGRs* were analyzed in three independent lines for each gene. It showed that in *SmHMGR2*-KO lines, the target gene SmHMGR2 was reduced compared with the wild-type. However, the expression level of SmHMGR1 was also reduced, along with significant upregulation of SmHMGR3 and SmHMGR5. SmHMGR4 showed no statistically significant upregulation, although it exhibited a slight overall increasing trend. Due to the low homology between the two genes (72.23% in total and 80% in the sgRNA), off-target effects are unlikely to happen in SmHMGR1. Thus, we proposed a regulatory network involving SmHMGR2 alongside SmHMGR1, SmHMGR3, and SmHMGR5 genes, and hypothesized that disruption of SmHMGR2 impaired the normal transcriptional activity of the HMGR family.

In the *SmHMGR5*-KO mutants, the target gene *SmHMGR5* displayed a slight downward trend, but without a statistically significant reduction. Correspondingly, the transcript levels of the other four homologous genes remained stable and showed no statistically significant changes (Supplementary Figure S3). These results indicate that the knockout of *SmHMGR2* can trigger the differential regulation of multiple homologous genes, whereas the knockout of *SmHMGR5* exhibits high functional specificity and has no obvious impact on the expression of other family members.

### 2.6. Comparative PLS-DA Analysis of SmHMGR2 and SmHMGR5 Knockout Mutants

To rapidly assess the effects of *SmHMGR2* and *SmHMGR5* genes on the secondary metabolites of *S. miltiorrhiza*, we treated the aforementioned mutants and wild-type plants with a combination elicitor of yeast extract (YE) combined with Ag^+^, which has been proven to rapidly induce the accumulation of tanshinones and salvianolic acids in *S. miltiorrhiza* [38]. In addition to each gene having 5 independent mutants, some lines also possess biological replicates. For instance, line 111-F12 has three biological replicates (111-F12, 111-F12-1, and 111-F12-2), along with the wild-type WT-7, which has three replicates, and PZK-9, which has two replicates.

**Figure 5:**
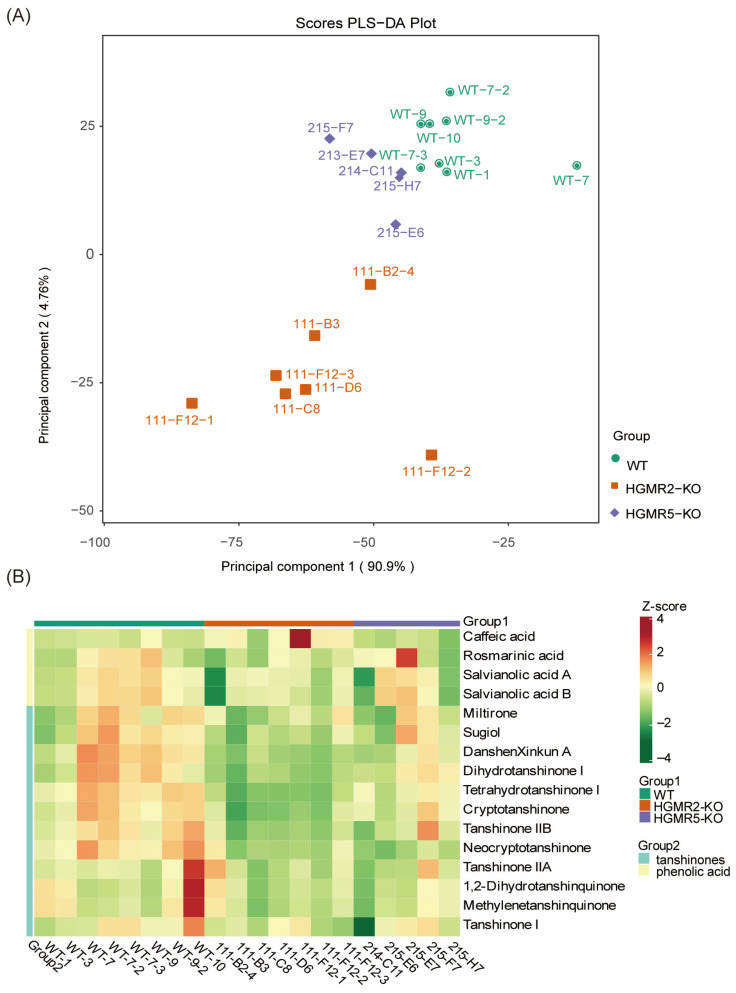
Multivariate statistical analysis of 15 target metabolites in WT, *SmHMGR2*-KO, and *SmHMGR 5*-KO hairy roots. (**A**) PLS-DA score plot of the samples. (**B**) Cluster heatmap of metabolite abundance based on Z-score normalization. The color scale represents the relative abundance of each metabolite across samples (red: high abundance; green: low abundance).

After 9 days of treatment, all samples were collected and analyzed using LC-QTOF-MS. The filtered data were then subjected to partial least squares discriminant analysis (PLS-DA) to visually display the differences in metabolic profiles among groups. It showed that the variance explained by the first principal component (PC1) and the second principal component (PC2) were 90.9% and 4.76%, respectively, with a cumulative explained variance of 95.36%. The model had an R^2^X of 0.956 and R^2^Y of 0.473, indicating the selected principal components could effectively reflect the main metabolic differences among samples. All wild-type (WT) samples were highly clustered in the range of PC1 (−40 to −20) and PC2 (10 to 35), whereas only WT-7 deviated slightly from the clustering center on the PC1 axis (PC1 ≈ −10). The *SmHMGR2*-KO mutants were mainly distributed in the range of PC1 (−85 to −35) and PC2 (−40 to 0), with relatively scattered clustering among mutant lines. Among them, the homozygous mutant 111-F12-2 deviated slightly on the PC1 axis (PC1 ≈ −35), suggesting a certain degree of line-specific variation in its metabolic profile (Figure 5A).

As for *SmHMGR5*-KO mutants, they were mainly distributed within the range of PC1 (−60 to −40) and PC2 (0 to 25), showing weaker separation from the WT group than the *SmHMGR2*-KO group, and the confidence intervals of some samples overlapped with the WT. This suggests that *SmHMGR5* knockout had a relatively mild disturbance on the metabolic profile (Figure 5A).

### 2.7. Analysis of Tanshinones and Salvianolic Acids in SmHMGR2 and SmHMGR5 Knockout Mutants

In order to further analyze the effect of *SmHMGR2* and *SmHMGR5* on tanshinone and phenolic acids, we identified 16 bioactive components (12 tanshinones and 4 phenolic acids) based on authentic chemical standards. A cluster heatmap was performed based on these 16 metabolites in each mutant (Supplementary Table S2), and it revealed that in the *SmHMGR2*-KO group, most of the tanshinone metabolites (e.g., cryptotanshinone, tanshinone II B, dihydrotanshinone I) displayed negative Z-scores (green), strongly indicating that loss of SmHMGR2 function exerted extensive and significant inhibitory effects on the biosynthesis of tanshinone compounds. This inhibitory effect was highly consistent between homozygous single-base insertion mutant lines (111-B3, 111-C8, 111-F12) and biallelic mutant lines (111-B2 and 111-D6), with no obvious line-specific differences observed, implying that the two mutation types caused comparable disruption of SmHMGR2 function (Figure 5B, Supplementary Table S3). Phenolic acid metabolites (rosmarinic acid, salvianolic acid B, and salvianolic acid A) showed slightly negative Z-scores (light green) across nearly all samples in the *SmHMGR2*-KO group compared with the WT group, with the exception of caffeic acid, as exemplified by rosmarinic acid (111-C8, Z = −1.0358), salvianolic acid A (111-F12-2, Z = −0.9319), and salvianolic acid B (111-B2-4, Z = −2.3822). This suggested that *SmHMGR2* knockout also inhibited the accumulation of these three phenolic acid components to a certain extent, although its effect was much weaker than that on tanshinones. However, caffeic acid was visualized as light yellow (Z-score > 0) in most *SmHMGR2*-KO samples, showing slightly increased accumulation (Supplementary Table S2). Notably, caffeic acid in 111-F12-1 was abnormally elevated compared with 111-F12-2 and 111-F12-3 (biological replicates from the same mutant).

As for *SmHMGR5*-KO mutants, the accumulation levels of tanshinone metabolites were intermediate between those of the WT and the *SmHMGR2*-KO group, mostly appearing light green or yellow (Z-score close to 0 or slightly negative). This indicates that the regulatory ability of *SmHMGR5* on tanshinone synthesis was significantly weaker than that of *SmHMGR2*. The overall changes in phenolic acid metabolites in the *SmHMGR5*-KO group were more moderate, with the Z-scores of most samples close to zero. A few samples even exhibited weak positive enrichment (light red), as seen for salvianolic acid B (e.g., 215-E6, Z = 1.1592). Notably, rosmarinic acid in 215-E7 was significantly higher than in other mutants (Z = 2.8616), further indicating that the regulatory effect of *SmHMGR5* on phenolic acid metabolism was extremely weak, with some lines showing no obvious inhibitory effect at all. Notably, the 10 bp deletion mutant 215-E7 and the single-base deletion mutant 215-F7 showed a lower degree of metabolic inhibition in the *SmHMGR5*-KO group, with the Z-scores of most tanshinones close to 0 and even positive enrichment of phenolic acids (Figure 5B). It is speculated that these two mutation types caused mild impairment of *SmHMGR5* function and may retain partial metabolic regulatory ability.

Because abnormal increases or decreases were observed in individual mutants, we subsequently treated *SmHMGR2*-KO, *SmHMGR5*-KO, and WT as three groups to analyze the changes in different components. We performed coefficient of variation (CV) analysis on *SmHMGR2*-KO and *SmHMGR5*-KO mutants to evaluate the stability of metabolic phenotypes across different lines. It showed that despite these quantitative variations, all independent lines displayed a consistent metabolic change pattern (Supplementary Table S3).

**Figure 6:**
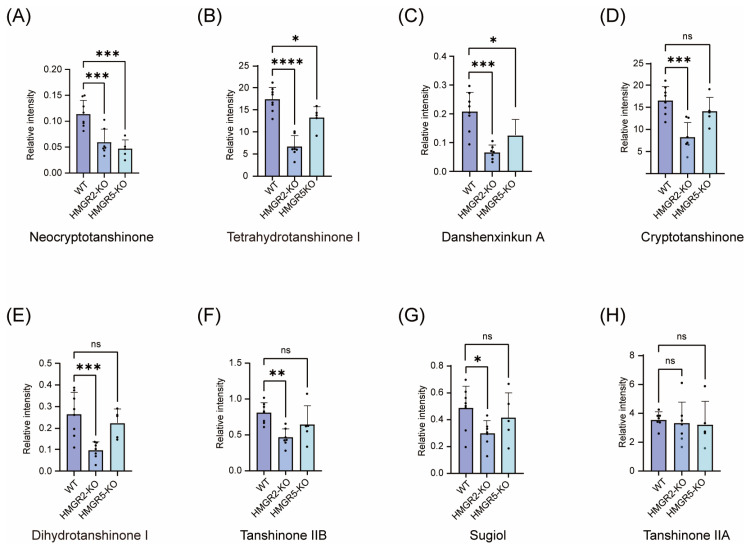
Effects of *SmHMGR2* and *SmHMGR5* knockout on the accumulation of representative tanshinones with significant differences in *S. miltiorrhiza* hairy roots. (**A**) Neocryptotanshinone; (**B**) Tetrahydrotanshinone I; (**C**) Danshenxinkun A; (**D**) Cryptotanshinone; (**E**) Dihydrotanshinone I; (**F**) Tanshinone IIB; (**G**) Sugiol; (**H**) Tanshinone IIA. Data are expressed as mean ± SD of biological replicates. Significance was determined by one-way ANOVA followed by Tukey’s post hoc test: * *p* < 0.05, ** *p* < 0.01, *** *p* < 0.001, **** *p* < 0.0001; ns, not significant (*p* > 0.05).

In *SmHMGR2*-KO mutants, danshenxinkun A showed the most substantial reduction, falling to 31.79% of WT levels, followed by dihydrotanshinone I (36.30%), tetrahydrotanshinone I (38.33%), tanshinone IIB (46.02%), cryptotanshinone (49.59%), and neocryptotanshinone (51.92%). Sugiol exhibited a moderate decrease to 60.77% of WT levels (Figure 6). In contrast, the levels of miltirone, tanshinone IIA, tanshinone I, methylenetanshinquinone, and 1,2-dihydrotanshinquinone did not change significantly (Supplementary Figure S4). In contrast to the broad inhibitory effect of *SmHMGR2* knockout, *SmHMGR5*-KO only significantly affected a small number of tanshinone components. In *SmHMGR5*-KO mutants, all five lines consistently exhibited downregulation of neocryptotanshinone, danshenxinkun A, and tetrahydrotanshinone I, decreasing to 40.79%, 60.17%, and 76.25% of WT levels, respectively, confirming the metabolic phenotype induced by *SmHMGR5* knockout (Figure 6). However, the contents of tanshinone IIB, dihydrotanshinone I, miltirone, tanshinone IIA, tanshinone I, 1,2-dihydrotanshinquinone, methylenetanshinquinone and other components showed no significant differences between mutants and WT (Supplementary Figure S4).

Despite occasional variations in individual lines (Supplementary Table S3), the phenolic acid components, including caffeic acid, rosmarinic acid, salvianolic acid A, and salvianolic acid B, were not significantly affected in both *SmHMGR2* and *SmHMGR5* knockout lines (Supplementary Figure S4).

## 3. Discussion

In this study, by integrating the annotation information of multiple versions of the *S. miltiorrhiza* reference genome and its transcriptome data, we successfully identified and cloned *SmHMGR5*, a novel member of the HMGR gene family, clarified that it belongs to the clade II with *SmHMGR2* and *SmHMGR3*, and located it in the adjacent region on chromosome 7 of the *S. miltiorrhiza* bh2-7 genome. Using the CRISPR/Cas9 gene editing technology, we constructed specific knockout mutants of *SmHMGR2* and *SmHMGR5*, and combined with UPLC-MS-based metabolomic analysis, we systematically elucidated the functional differences and molecular mechanisms of the two genes in the regulation of secondary metabolism in *S. miltiorrhiza*.

**Figure 7:**
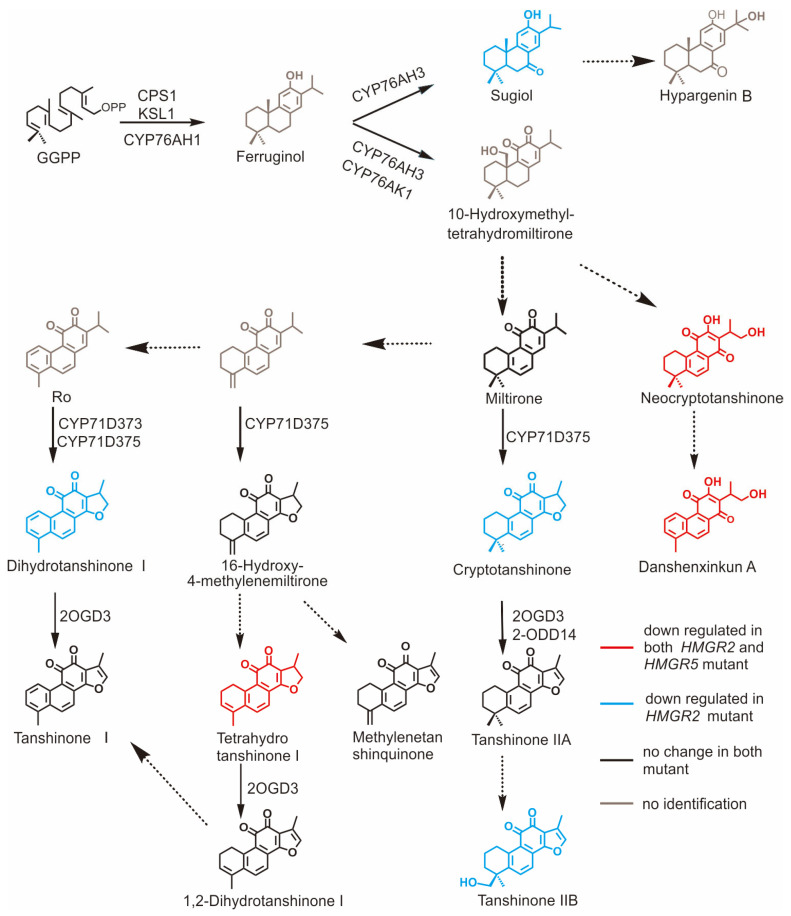
Impact of *SmHMGR2* and *SmHMGR5* mutations on the simplified tanshinone biosynthesis pathway. Solid arrows represent characterized biosynthetic pathways and their corresponding enzymes; dashed arrows represent putative pathways. Colored compounds exhibit significantly altered accumulation in the mutants: red denotes metabolites downregulated in both *SmHMGR2* and *SmHMGR5* mutants, while blue indicates those downregulated exclusively in the *SmHMGR2* mutant. Black compounds show no significant changes in either mutant line. Gray arrows point to unmeasured intermediates.

As a key rate-limiting enzyme in the MVA pathway, loss of *SmHMGR2* function exerted a broad and strong inhibitory effect on tanshinone biosynthesis, with the contents of cryptotanshinone, neocryptotanshinone, danshenxinkun A, and other components decreasing by 30–60% (Figure 7). In contrast, it had a weak effect on the accumulation of phenolic acid metabolites, and the contents of caffeic acid, rosmarinic acid, salvianolic acid A and salvianolic acid B showed no significant changes before and after the gene knockout, suggesting that its regulatory role was mainly concentrated in the MVA pathway with limited interference from the phenylpropanoid pathway. In comparison, *SmHMGR5* had a milder regulatory effect on the secondary metabolism of *S. miltiorrhiza*, only significantly inhibiting the synthesis of neocryptotanshinone, danshenxinkun A and tetrahydrotanshinone I, and the contents of other tanshinones and all phenolic acid metabolites had no obvious changes, indicating that *SmHMGR5* undertakes an auxiliary function in the metabolic regulatory network, and its role can be partially compensated by other members of the family.

### 3.1. Stepwise Bottleneck Regulation of the Tanshinone Biosynthetic Pathway and Catalytic Properties of Sm2OGD3

In addition to the above significantly decreased compounds, the contents of some compounds in the tanshinone biosynthetic pathway, such as miltirone, tanshinone IIA, methylenetanshinquinone, 1,2-dihydrotanshinquinone, and tanshinone I, remained stable in both types of mutants (Figure 7). This phenomenon is highly consistent with the effect of SmHMGR1 and SmHMGR2 overexpression on tanshinones [20,23]. This stability is presumably associated with the catalytic activity of the key enzymes responsible for the biosynthesis of these compounds, which exhibit relatively low enzymatic activity and thus require a sufficiently high substrate concentration to trigger their significant catalytic effects. Sm2OGD3 is the key enzyme mediating the C-15,16 dehydrogenation of tanshinones. Sm2OGD3 can specifically catalyze the conversion of cryptotanshinone, 15,16-dihydrotanshinone I, and 15,16-tetrahydrotanshinone I to tanshinone IIA, tanshinone I, and 1,2-dihydrotanshinquinone, respectively, which is a key node regulating the biosynthesis of these three important tanshinones [17].

Pan et al. proposed that although Sm2OGD3 has a higher substrate affinity and catalytic efficiency for tetrahydrotanshinone I than cryptotanshinone in vitro, the content of cryptotanshinone is much higher than that of tetrahydrotanshinone I in *S. miltiorrhiza* plants, and its product (tanshinone IIA) is also correspondingly higher. This indicates that such enzymes have inherently low catalytic efficiency, and their in vivo conversion efficiency may be more dependent on the actual substrate concentration rather than simply being determined by the affinity between the enzyme and its substrate [17]. Therefore, this also aptly explains why the content of tanshinone IIA was not affected by the gene knockout and did not decrease against the background of the reduced content of cryptotanshinone after *SmHMGR2* knockout. A study by Kai et al. also provided strong evidence: overexpression of SmHMGRs or SmGGPPS alone failed to significantly increase the yield of tanshinone IIA, while their synergistic co-expression significantly promoted the accumulation of both cryptotanshinone and tanshinone IIA. This indicates that the high-flux conversion potential of this node can only be activated when the upstream flux is fully enhanced and the substrate concentration breaks through the threshold of the node. In this experiment, Sm2OGD3 in other nodes of the catalytic network also showed similar regulatory characteristics [20]. After the knockout of *SmHMGR2* and *SmHMGR5*, the decrease in the content of tetrahydrotanshinone I did not cause a reduction in its product, 1,2-dihydrotanshinquinone. Meanwhile, the decrease in the content of dihydrotanshinone I also did not lead to a significant change in its product tanshinone I. This further supports that Sm2OGD3 may be a type of enzyme with low catalytic efficiency and high sensitivity to substrate concentration. The above results indicate that the conversion from cryptotanshinone to tanshinone IIA is a key node in the tanshinone synthesis network that is strictly regulated by enzyme activity and substrate concentration. Sm2OGD3, which catalyzes this step, has limited efficiency under normal metabolic conditions and is insensitive to fluctuations in upstream flux, which explains the stability of tanshinone IIA after the knockout of *SmHMGR2* and *SmHMGR5*, but after the upstream pathway is fully enhanced, this node shows the ability to perform high-flux conversion, which is also consistent with the synergistic enhancement phenomenon in co-expression experiments, reflecting the “stepwise bottleneck” regulatory mechanism in the tanshinone synthesis network.

Although the content of tanshinone IIA remained stable after *SmHMGR2* knockout, the content of its downstream product, tanshinone IIB, was significantly reduced. This phenomenon suggests that the unknown enzymes catalyzing this conversion step may have high catalytic efficiency and that their activity is highly sensitive to changes in substrate concentration. Although the total amount of tanshinone IIA remained stable, the “effective tanshinone IIA pool” available for downstream conversion may have decreased, and the effective concentration of tanshinone IIA may have fallen below the kinetic threshold for the efficient catalysis of these enzymes, thus leading to the blockage of tanshinone IIB synthesis and a decrease in its content. This finding not only deepens the understanding of the dynamic regulatory mechanism of the tanshinone biosynthetic pathway but also provides a clear direction for metabolic engineering strategies. In the future, while optimizing the upstream flux, attention should be paid to the catalytic efficiency of such key node catalytic enzymes [39], and the yield of target products should be effectively improved through a multi-target synergistic strategy.

### 3.2. Metabolic Flux Redirection and Precursor Competition Regulation in the Salvianolic Acid Biosynthetic Pathway

Tanshinones are mainly synthesized via the MVA and MEP pathways, while salvianolic acids are derived from the phenylpropanoid pathway. These two pathways share intracellular precursor resources such as ATP, NADPH, and CoA [40]. The corresponding metabolic flux redistribution and precursor competition mechanism are illustrated in Supplementary Figure S5. Based on the cluster analysis results, we observed that *SmHMGR2* knockout led to a slight increase in caffeic acid content, accompanied by a mild decrease in other salvianolic acid components, and we speculate that the inhibition of the MVA pathway may upregulate the activity of the phenylpropanoid pathway through an unknown signaling mechanism, driving the redistribution of shared precursors toward this branch [3,41,42,43].

This change may further induce competition between the two downstream synthetic branches: the pathway using p-coumaroyl-CoA as the acyl donor, and the pathway using caffeoyl-CoA as the acyl donor. Since these two branches share p-coumaric acid as a common precursor, the accumulation of caffeic acid may simultaneously inhibit the conversion of p-coumaroyl-CoA to downstream products such as rosmarinic acid, particularly the esterification step [2,44,45]. Notably, caffeic acid can be converted to the acyl donor caffeoyl-CoA via specific catalytic reactions, which can then undergo esterification with acyl acceptors such as 4-hydroxyphenyllactic acid or danshensu to generate rosmarinic acid precursors [45,46]. Although the content of caffeoyl-CoA was not determined in this study, our current results suggest that the downstream esterification reaction using caffeoyl-CoA as the acyl donor may not be the dominant route for rosmarinic acid precursor synthesis. Instead, the esterification reaction using p-coumaroyl-CoA as the acyl donor remains the primary pathway in this process.

### 3.3. Functional Differentiation and Metabolic Regulation of the SmHMGR Gene Family Based on Transcriptional Changes and Evolutionary Characteristics

Phylogenetic and collinearity analyses revealed that SmHMGR1 and SmHMGR4 belong to one clade, whereas SmHMGR2, SmHMGR3, and SmHMGR5 fall into a separate clade, suggesting that these homologs have undergone substantial functional divergence during evolution.

In *SmHMGR2*-KO mutant lines, transcript levels of SmHMGR1 and SmHMGR2 were significantly reduced, whereas SmHMGR5 was significantly upregulated; SmHMGR3 and SmHMGR4 exhibited a slight upward trend with no significant difference. Despite belonging to distinct evolutionary clades, SmHMGR1 and SmHMGR2 displayed a co-downregulated expression pattern, implying that they are coordinately controlled by the core feedback regulatory network of the MVA pathway regardless of their separate evolutionary origins. The marked upregulation of SmHMGR5 indicates that *SmHMGR2* knockout elicits a compensatory response within the SmHMGR, and SmHMGR5 may partially compensate for the catalytic function of SmHMGR2 in the MVA pathway. Nevertheless, metabolomic data showed that the contents of cryptotanshinone, neocryptotanshinone, danshenxinkun A, and other tanshinones remained significantly decreased, demonstrating that the compensatory effect of SmHMGR5 is limited and insufficient to fully substitute the core regulatory role of SmHMGR2 in tanshinone biosynthesis.

In *SmHMGR5*-KO mutant lines, the transcript level of SmHMGR5 showed a slight downward trend without statistical significance, and the expression levels of the other four homologs remained stable. This result indicates that *SmHMGR5* knockout possesses high functional specificity and does not trigger obvious transcriptional feedback regulation of other family members. Consistently, metabolomic analysis showed that only three tanshinone compounds (neocryptotanshinone, danshenxinkun A, and tetrahydrotanshinone I) were significantly downregulated, whereas other tanshinones and all phenolic acids were unaffected. Such a mild and specific metabolic phenotype is highly consistent with the expression profile of the SmHMGRs, further verifying that SmHMGR5 only serves an auxiliary and fine-tuning function in the tanshinone biosynthetic pathway.

Collectively, SmHMGR2 and SmHMGR5 exhibit clear hierarchical functional differentiation: SmHMGR2 acts as a core regulator that globally governs tanshinone biosynthesis, and its loss triggers a family-wide compensatory transcriptional response but cannot reverse the overall repressive metabolic phenotype. In contrast, SmHMGR5 functions as a specific auxiliary regulator whose deletion exerts no notable influence on the transcription of other homologs and affects only a limited number of downstream metabolites. The SmHMGRs thus forms a coordinately regulated and functionally complementary regulatory module in the MVA pathway, which collectively sustains the metabolic homeostasis of tanshinone biosynthesis. These findings provide key transcriptional evidence for interpreting the divergent metabolic phenotypes between the two mutant lines.

## 4. Materials and Methods

### 4.1. Plant Materials and Strains

The plant material used in this experiment was the sixth-generation inbred line bh2-7 of *S. miltiorrhiza*. Aseptic seedlings were cultured on 1/2 MS medium (Murashige and Skoog medium, pH 5.8) under controlled conditions at 25 °C with a photoperiod of 16 h light/8 h dark. Tissue culture seedlings of *Salvia miltiorrhiza* that had been subcultured for approximately one month, with normal rooting and good growth status, were selected as the material for hairy root induction. *Agrobacterium rhizogenes* C58C1 (competent cells) was prepared in the laboratory.

### 4.2. RNA Extraction, Reverse Transcription, and Cloning of Full-Length cDNA of SmHMGR5

Fresh leaves of *S. miltiorrhiza* bh2-7 were collected, and total RNA was extracted using the RNAeasy Fast Plant Total RNA Extraction Kit (Tiangen Biotech, Beijing, China). Subsequently, total RNA was reverse-transcribed into cDNA using the FastKing First-Strand cDNA Synthesis Kit (gDNA Eraser) (Tiangen Biotech, Beijing, China). Primers were designed using SnapGene 6.0.2 software: gene cloning primers were designed based on the *S. miltiorrhiza* bh2-7 reference genome (Detailed information on the bh2-7 reference genome is shown in Supplementary Table S1), while the homologous recombination primers used for vector construction consisted of approximately 20 bp of vector homology arm, a restriction site, and approximately 20 bp of target gene-specific sequence. First, the full-length cDNA of *SmHMGR5* was amplified using gene cloning primers, followed by PCR amplification based on the cDNA using homologous recombination primers. PCR products were purified and ligated into the KpnIsite within MCS1 of the pC1300-TP2-P19 vector prior to sequencing [47]. Gene cloning primers and homologous recombination primers for vector construction are shown in Supplementary Table S4.

### 4.3. Quantitative Real-Time PCR (qRT-PCR) Analysis

To determine the transcript levels of *SmHMGR*1–5 in gene-edited mutant lines and wild-type hairy roots, quantitative real-time PCR (qRT-PCR) was conducted. Total RNA was extracted from hairy root samples using the RNAeasy Fast Plant Total RNA Extraction Kit (Tiangen Biotech, Beijing, China), and first-strand cDNA was synthesized using Hifair^®^ AdvanceFast One-step RT-gDNA Digestion SuperMix for qPCR (11151ES60, Yeasen, Shanghai, China) following the manufacturer’s instructions. The qRT-PCR reaction was performed with TB Green^®^ Premix Ex Taq™ II (Tli RNaseH Plus) (RR420A, TaKaRa Biotech, Kusatsu, Japan) on a LightCycler 480 System (Roche, Basel, Switzerland) using the specific primers listed in Supplementary Table S4. The PCR program was set as follows: 95 °C for 30 s, followed by 40 cycles of 95 °C for 5 s and 60 °C for 30 s, with a melting curve analysis from 60 °C to 95 °C to verify amplification specificity. Gene expression levels were calculated using the 2^−ΔΔCt^ method with *SmActin* as the internal reference gene, and three independent biological replicates and three technical replicates were set for each sample.

### 4.4. Bioinformatics Analysis of SmHMGRs

The amino acid sequences of *SmHMGR3* (JN831102.1), cloned from an unknown *S. miltiorrhiza* line, and *SmHMGR3* and *SmHMGR5* (this study; line bh2-7) were selected for multiple sequence alignment using the ClustalW algorithm in MEGA 12.0 software. The alignment results were visualized using the ESPript 3.0 tool. The theoretical isoelectric point and molecular weight were predicted using the Compute pI/MW tool on the ExPASy server (https://web.expasy.org/compute_pi/; accessed on 24 April 2025); the transmembrane domains were analyzed using DeepTMHMM-1.0 (DeepTMHMM 1.0—DTU Health Tech—Bioinformatic Services). To elucidate the evolutionary characteristics of the HMGR family genes in *Lamiaceae*, to which *S. miltiorrhiza* belongs, this study performed a gene synteny analysis using the GenoCGLLM platform (a gene cluster analysis tool based on comparative genomics and large language models, https://www.bic.ac.cn/SynCoIV/; accessed on 24 April 2025) [34]. The maximum likelihood (ML) phylogenetic tree was constructed using MEGA software based on the 41 HMGR protein sequences from 11 species [48]. The standard bootstrap method was used to evaluate the reliability of branches with 1000 bootstrap replicates. The Jones-Taylor-Thornton (JTT) model was selected as the amino acid substitution model.

### 4.5. Vector Construction

Based on the vector design strategy of the high-efficiency *Lactuca sativa* genome editing system, the pZKD672 vector targeting the *SmHMGR2* and *SmHMGR5* genes of *S. miltiorrhiza* was constructed in this study. The vector was constructed using pKSE401 as the backbone and was optimized through the intron-mediated enhancement (IME) strategy, with the 2 × 35S promoter driving the co-expression of SpCas9 and sgRNA [37]. The sgRNA target sequences were designed using the online CRISPOR tool (https://crispor.gi.ucsc.edu/crispor.py; accessed on 27 April 2025). Three 20 bp guide sequences were designed in this study (sgRNA-111: 5′-TCTACTTTCTCCTCCTCCGC-3′; sgRNA-214: 5′-GTCGCCGGTGCAGTTCTCGC-3′; sgRNA-215: 5′-CTAAATCTGCTGGGGGTGAA-3′), and each sequence was cloned into an engineered intron containing a tRNA structure. The pZKD672 vector was digested with BsaI, and a 15 kb fragment was obtained by gel purification. The forward and reverse target-specific primers were annealed (95 °C for 30 s, 50 °C for 30 s, then kept at 4 °C). In a 10 μL reaction system, the annealed primers were ligated with the purified vector fragment at 16 °C overnight. The reaction system contained 1 μL 10 × T4 DNA Ligase Buffer, 2 μL BsaI-digested linearized vector, 5 μL annealed primers, 0.5 μL T4 DNA Ligase and 2 μL ddH_2_O. The ligation product was transformed into *Escherichia coli* DH5α competent cells, and colonies were screened on LB agar plates containing kanamycin. Colony PCR was performed using target-specific primers and R50 primer (R50 primer sequence: 5′-tgtcgaacaggagagcgcca-3′) to identify clones containing the desired insert. Positive clones were selected for plasmid extraction and sequencing to confirm the correct insertion of the target sequence.

### 4.6. Hairy Root Transformation and Identification of Positive Lines

#### 4.6.1. Establishment of Transformation System

*Agrobacterium rhizogenes* C58C1 was used to induce hairy root formation in *S. miltiorrhiza* line bh2-7 via the leaf disk transformation method. The bacterial solution of the successfully constructed gene editing vector pZKD672 was cultured to an OD_600_ of 0.6, then resuspended in MS liquid medium and used to soak sterile leaf explants (1 cm^2^) for 5 min. After absorbing the excess bacterial solution with sterile filter paper, the explants were co-cultured at 25 °C in the dark for 2 days. Subsequently, the explants were transferred to 1/2 MS selective solid medium containing 200 mg L^−1^ Timentin to induce hairy root formation.

#### 4.6.2. DNA Extraction and Target Site Amplification

Genomic DNA was extracted from approximately 2 mg of a single hairy root clone using the Plant Genomic DNA Extraction Kit (AK101, Biomed Biotech, Beijing, China). Specific primers were designed for PCR amplification targeting the editing sites of *SmHMGR2* and *SmHMGR5* (covering approximately 200 bp upstream and downstream of each target sequence). The 20 μL PCR reaction system contained 10 μL KOD One™ PCR Master Mix -Blue, 0.5 μM each of upstream and downstream primers (*SmHMGR2*-F: 5′-CGTGGTCATATATGGGAGTGA-3′), (*SmHMGR2*-R: 5′-GCTCAGCGGGTAACATCTT-3′); (*SmHMGR5*-F: 5′-GCATGCTAGCAACATCGTCTCG-3′), (*SmHMGR5*-R: 5′-GTTGTTTGTTAAGTGATGGTACCC-3′), 2 μL DNA template, and was supplemented with ddH_2_O to the final volume. The amplification program was as follows: initial denaturation at 98 °C for 3 min; denaturation at 98 °C for 10 s, annealing at 55 °C for 15 s, extension at 68 °C for 10 s (35 cycles); final extension at 68 °C for 5 min.

#### 4.6.3. Mutant Detection

The PCR products were subjected to 1.5% agarose gel electrophoresis run in 1 × TAE buffer (220 V for 12 min). Potential mutant samples were initially screened by comparing their bands with those of wild-type hairy roots. Positive bands were purified and sent to the sequencing company for Sanger sequencing. The mutation types were analyzed using Synthego ICE software (EditCo—CRISPR Performance Analysis). Before analyzing the editing results, samples with a Model Fit (R^2^) value less than 80% were excluded. Lines with an insertion/deletion efficiency greater than 20% were defined as successfully edited lines. Among them, homozygous mutants showed a single mutant peak, biallelic mutants showed overlapping double peaks, and chimeric mutants showed mixed multiple peaks.

#### 4.6.4. Off-Target Verification

Due to the high sequence homology between *SmHMGR3* and *SmHMGR5*, potential off-target effects were examined in sgRNA-215-mediated mutant lines. Five independent lines used for metabolic analysis (214-C11, 215-E6/E7/F7/H7) were selected for off-target detection. PCR amplification was performed using the SmHMGR3-specific primers 39001-F (5′-TTGCATTGAGGTAGGGACAGTA-3′) and 39001-R (5′-ATGCTCGCAACATCCTTATT-3′). The amplified products were subjected to Sanger sequencing in the reverse direction using primer 39001-R. If the *SmHMGR3* gene is not edited in these mutants, it indicates that sgRNA-215 can specifically target *SmHMGR5* without off-target editing at its homologous sequence *SmHMGR3*.

### 4.7. Metabolite Analysis

#### 4.7.1. Sample Preparation and Metabolite Extraction

The homozygous mutant hairy roots screened in Section 2.4 were subcultured on 1/2 MS solid medium at 25 °C in the dark for 1 month. Subsequently, the hairy roots were transferred to solid induction medium supplemented with 100 μM AgNO_3_ (silver ion) and 2500 mg/L yeast extract, which have been reported to effectively enhance the accumulation of tanshinones [49,50]. After 9 days of further cultivation, the samples were collected, freeze-dried for 48 h and ground into fine powder, and stored at −80 °C for later use.

For metabolite extraction, 20 mg of freeze-dried powder was accurately weighed into a 2 mL centrifuge tube, and 1.5 mL of 70% (*v*/*v*) methanol aqueous solution containing berberine (2 μg/mL) as an internal standard was added. After vortex oscillation for 60 s, ultrasonic extraction was performed at low temperature for 45 min. The extract was centrifuged at 4 °C and 12,000 rpm for 10 min, and the supernatant was transferred to a new 2 mL centrifuge tube and incubated at −20 °C for 1 h to precipitate proteins. Then centrifugation was performed under the same conditions, and the supernatant was collected and transferred to an HPLC vial for ultra-performance liquid chromatography-mass spectrometry (UPLC-MS) analysis.

#### 4.7.2. Chromatographic and Mass Spectrometric Conditions

Chromatographic conditions: A Waters Acquity UPLC column (100 × 2.1 mm, 1.8 μm) was used. The column temperature was set at 40 °C, with a flow rate of 0.4 mL/min and an injection volume of 1 μL. The mobile phase consisted of ultrapure water (A) and acetonitrile (B), both containing 0.1% formic acid. The gradient elution program was as follows: 0–1 min, 10% B; 1–1.5 min, 10% to 18% B; 1.5–3 min, 18% B; 3–3.5 min, 18% to 22% B; 3.5–4 min, 22% to 30% B; 4–5.5 min, 30% to 38% B; 5.5–7 min, 38% to 45% B; 7–15 min, 45% to 60% B; 15–17.5 min, 60% to 70% B; 17.5–19.5 min, 70% to 90% B; 19.5–20.5 min, 90% to 10% B; 20.5–23 min, 10% B. During the entire analysis, the samples were kept at 4 °C in the autosampler. To minimize the influence of instrumental signal fluctuations, the samples were injected continuously in a random order. Quality Control (QC) samples were inserted evenly in the sample analysis sequence to monitor and evaluate the system stability and the reliability of experimental data.

Mass spectrometric conditions: An electrospray ionization (ESI) source was used for positive ion mode scanning. The scanning range was set at *m*/*z* 50–1200 Da, the ion source temperature was 120 °C, and the desolvation gas temperature was 450 °C. The desolvation gas flow rate was set at 900 L/h, the scanning time was 0.15 s, and the total detection time was 23 min. The collision energy range was 50 to 80 V, and the cone voltage was 40 V. Nitrogen was used as the desolvation gas. Real-time mass correction was performed using leucine enkephalin (200 pg·L^−1^), and the entire process was intelligently controlled by Masslynx 4.1 software.

#### 4.7.3. Data Processing

The original data were preprocessed using Progenesis QI v3.0 (Waters Corporation, Milford, MA, USA) software. Upon data import, the “Start automatic processing” function was enabled, and the software sequentially performed baseline filtering, peak identification, peak matching, retention time correction, and peak alignment. Specifically, the software automatically selected the run with the smallest overall difference among all samples as the alignment reference and performed nonlinear retention time correction to ensure accurate matching of the same compound across different samples. Peak identification was carried out using the “automatic sensitivity” mode, detecting both high- and low-abundance ion features, and adduct ions (e.g., [M+H]^+^, [M-H]^−^, [M+Na]^+^) were automatically deconvoluted to improve quantification accuracy. Following automatic processing, a data matrix containing retention time, mass-to-charge ratio (*m*/*z*), and normalized abundance was obtained.

To reduce data noise, zero-value filtering was applied to the data matrix, eliminating metabolites with more than 80% zero values across all samples to avoid the impact of missing values on subsequent statistical analysis. The filtered data were normalized by the peak area of berberine as an internal standard, i.e., the abundance of each metabolite in each sample was divided by the peak area of berberine in the corresponding sample, to correct for systematic variations caused by sample loading amount, instrument response, and other factors between samples. 

#### 4.7.4. Selection of Target Metabolites

SmHMGRs are key rate-limiting enzyme genes in the mevalonate (MVA) pathway of *S. miltiorrhiza*. Meanwhile, the metabolic flux regulated by them shares precursors (such as acetyl-CoA and NADPH) with the phenylpropanoid pathway. Therefore, gene knockout will directly affect the synthesis of downstream tanshinones (derived from the MVA pathway) and phenolic acids (derived from the phenylpropanoid pathway). Based on this, this study focused on such metabolites and extracted metabolic data of 15 important active components by referring to relevant reference information, including tanshinones (Class_ I): Miltirone, Tanshinone IIB, Danshenxinkun A, Dihydrotanshinone I, Tetrahydrotanshinone I, Neocryptotanshinone, Tanshinone I, Sugiol, 1,2-Dihydrotanshinquinone, Methylenetanshinquinone, Tanshinone IIA, Cryptotanshinone; phenolic acids (Class_II): Caffeic acid, Rosmarinic acid, Salvianolic acid A, Salvianolic acid B.

In addition to the above 16 targeted metabolites, untargeted global metabolic profiling was also conducted in this study. The full lists of significantly upregulated and downregulated differential metabolites in *SmHMGR2*-KO and *SmHMGR5*-KO lines relative to wild-type controls are summarized in Supplementary Table S5.

#### 4.7.5. Metabolite Data Analysis

To clarify the differential accumulation characteristics of the 16 target metabolites in wild-type (WT), *SmHMGR2* knockout (*SmHMGR2*-KO), and *SmHMGR5* knockout (*SmHMGR5*-KO) hairy roots. Supervised partial least squares discriminant analysis (PLS-DA) was performed using Metware Cloud online software (https://cloud.metware.cn; accessed on 17 October 2025) on the normalized data matrix to reveal the overall distribution differences in metabolites between groups. Meanwhile, a cluster heatmap was drawn based on Z-score normalization to intuitively show the abundance patterns of the 15 metabolites in different samples. Finally, GraphPad Prism 10.1.2 software was used to perform inter-group comparison and significance test (e.g., *t*-test or analysis of variance, ANOVA) of the accumulation of the 16 metabolites among the WT, *SmHMGR2*-KO and *SmHMGR5*-KO groups, so as to evaluate the specific effects of gene knockout on the synthesis of various metabolites and locate the core regulated target metabolites.

## 5. Conclusions

In this study, based on genome annotations from multiple *Salvia miltiorrhiza* reference assemblies and transcriptome data, we successfully identified and cloned *SmHMGR5*, a novel member of the HMGR gene family. Using CRISPR/Cas9 gene editing, we generated knockout mutants of *SmHMGR2* and *SmHMGR5*. We demonstrated that knockout of *SmHMGR2* caused broad and strong inhibition of tanshinone biosynthesis, whereas knockout of *SmHMGR5* only affected the accumulation of select tanshinone compounds. We further discussed the stepwise bottleneck regulation at downstream catalytic nodes in the tanshinone biosynthetic pathway triggered by the deficiency of these two upstream genes. This work provides theoretical support and technical guidance for unraveling the precise regulatory network of tanshinone biosynthesis and for optimizing the accumulation of medicinal components in *S. miltiorrhiza* via multi-target synergistic metabolic engineering.

## Data Availability

All the data supporting the findings of this study are available in the paper and Supplementary Data.

## Supplementary Materials

The following supporting information can be downloaded at: https://www.mdpi.com/article/10.3390/ijms27083485/s1.

## References

[1] Ren J., Fu L., Nile S.H., Zhang J., Kai G. (2019). *Salvia miltiorrhiza* in Treating Cardiovascular Diseases: A Review on Its Pharmacological and Clinical Applications. Front. Pharmacol..

[2] Lu S. (2021). Biosynthesis and Regulatory Mechanisms of Bioactive Compounds in *Salvia miltiorrhiza*, a Model System for Medicinal Plant Biology. Crit. Rev. Plant Sci..

[3] Xu Z., Ji A., Zhang X., Song J., Chen S. (2016). Biosynthesis and Regulation of Active Compounds in Medicinal Model Plant *Salvia miltiorrhiza*. Chin. Herb. Med..

[4] Jiang Z., Gao W., Huang L. (2019). Tanshinones, Critical Pharmacological Components in *Salvia miltiorrhiza*. Front. Pharmacol..

[5] Chen X., Yu J., Zhong B., Lu J., Lu J.-J., Li S., Lu Y. (2019). Pharmacological Activities of Dihydrotanshinone I, a Natural Product from *Salvia miltiorrhiza* Bunge. Pharmacol. Res..

[6] Fang J., Little P.J., Xu S. (2018). Atheroprotective Effects and Molecular Targets of Tanshinones Derived from Herbal Medicine Danshen. Med. Res. Rev..

[7] Ke L., Zhong C., Chen Z., Zheng Z., Li S., Chen B., Wu Q., Yao H. (2023). Tanshinone I: Pharmacological Activities, Molecular Mechanisms against Diseases and Future Perspectives. Phytomedicine.

[8] Chien M.-Y., Chuang C.-H., Chern C.-M., Liou K.-T., Liu D.-Z., Hou Y.-C., Shen Y.-C. (2016). Salvianolic Acid A Alleviates Ischemic Brain Injury through the Inhibition of Inflammation and Apoptosis and the Promotion of Neurogenesis in Mice. Free Radic. Biol. Med..

[9] Qin T., Rasul A., Sarfraz A., Sarfraz I., Hussain G., Anwar H., Riaz A., Liu S., Wei W., Li J. (2019). Salvianolic Acid A & B: Potential Cytotoxic Polyphenols in Battle against Cancer via Targeting Multiple Signaling Pathways. Int. J. Biol. Sci..

[10] He Y., Lu R., Wu J., Pang Y., Li J., Chen J., Liu B., Zhou Y., Zhou J. (2020). Salvianolic Acid B Attenuates Epithelial-Mesenchymal Transition in Renal Fibrosis Rats through Activating Sirt1-Mediated Autophagy. Biomed. Pharmacother..

[11] Wang Z., Peters R.J. (2022). Tanshinones: Leading the Way into Lamiaceae Labdane-Related Diterpenoid Biosynthesis. Curr. Opin. Plant Biol..

[12] Gao W., Hillwig M.L., Huang L., Cui G., Wang X., Kong J., Yang B., Peters R.J. (2009). A Functional Genomics Approach to Tanshinone Biosynthesis Provides Stereochemical Insights. Org. Lett..

[13] Cui G., Duan L., Jin B., Qian J., Xue Z., Shen G., Snyder J.H., Song J., Chen S., Huang L. (2015). Functional Divergence of Diterpene Syntheses in the Medicinal Plant *Salvia miltiorrhiza* Bunge. Plant Physiol..

[14] Guo J., Ma X., Cai Y., Ma Y., Zhan Z., Zhou Y.J., Liu W., Guan M., Yang J., Cui G. (2016). Cytochrome P450 Promiscuity Leads to a Bifurcating Biosynthetic Pathway for Tanshinones. New Phytol..

[15] Guo J., Zhou Y.J., Hillwig M.L., Shen Y., Yang L., Wang Y., Zhang X., Liu W., Peters R.J., Chen X. (2013). CYP76AH1 Catalyzes Turnover of Miltiradiene in Tanshinones Biosynthesis and Enables Heterologous Production of Ferruginol in Yeasts. Proc. Natl. Acad. Sci. USA.

[16] Ma Y., Cui G., Chen T., Ma X., Wang R., Jin B., Yang J., Kang L., Tang J., Lai C. (2021). Expansion within the CYP71D Subfamily Drives the Heterocyclization of Tanshinones Synthesis in *Salvia miltiorrhiza*. Nat. Commun..

[17] Pan X., Chang Y., Li C., Qiu X., Cui X., Meng F., Zhang S., Li X., Lu S. (2023). Chromosome-Level Genome Assembly of *Salvia miltiorrhiza* with Orange Roots Uncovers the Role of Sm2OGD3 in Catalyzing 15,16-Dehydrogenation of Tanshinones. Hortic. Res..

[18] Ma Y., Yuan L., Wu B., Li X., Chen S., Lu S. (2012). Genome-Wide Identification and Characterization of Novel Genes Involved in Terpenoid Biosynthesis in *Salvia miltiorrhiza*. J. Exp. Bot..

[19] Liao P., Zhou W., Zhang L., Wang J., Yan X., Zhang Y., Zhang R., Li L., Zhou G., Kai G. (2009). Molecular Cloning, Characterization and Expression Analysis of a New Gene Encoding 3-Hydroxy-3-Methylglutaryl Coenzyme A Reductase from *Salvia miltiorrhiza*. Acta Physiol. Plant..

[20] Kai G., Xu H., Zhou C., Liao P., Xiao J., Luo X., You L., Zhang L. (2011). Metabolic Engineering Tanshinone Biosynthetic Pathway in *Salvia miltiorrhiza* Hairy Root Cultures. Metab. Eng..

[21] Shi M., Luo X., Ju G., Yu X., Hao X., Huang Q., Xiao J., Cui L., Kai G. (2014). Increased Accumulation of the Cardio-Cerebrovascular Disease Treatment Drug Tanshinone in *Salvia miltiorrhiza* Hairy Roots by the Enzymes 3-Hydroxy-3-Methylglutaryl CoA Reductase and 1-Deoxy-d-Xylulose 5-Phosphate Reductoisomerase. Funct. Integr. Genom..

[22] Majewska M., Szymczyk P., Gomulski J., Jeleń A., Grąbkowska R., Balcerczak E., Kuźma Ł. (2022). The Expression Profiles of the *Salvia miltiorrhiza* 3-Hydroxy-3-Methylglutaryl-Coenzyme A Reductase 4 Gene and Its Influence on the Biosynthesis of Tanshinones. Molecules.

[23] Dai Z., Cui G., Zhou S.-F., Zhang X., Huang L. (2011). Cloning and Characterization of a Novel 3-Hydroxy-3-Methylglutaryl Coenzyme A Reductase Gene from *Salvia miltiorrhiza* Involved in Diterpenoid Tanshinone Accumulation. J. Plant Physiol..

[24] Yao Q., Ye Y., Yu M., Tian Y., Liu Q., Zheng H., Huang L. (2025). Engineering Prime Editors in *Salvia miltiorrhiza* for Precise Genome Modification. J. Integr. Plant Biol..

[25] Tian M., Luo L., Jin B., Liu J., Chen T., Tang J., Shen Y., Zhang H., Guo J., Zhang H. (2025). Highly Efficient *Agrobacterium rhizogenes*-mediated Gene Editing System in *Salvia miltiorrhiza* Inbred Line Bh2-7. Plant Biotechnol. J..

[26] Xu H., Song J., Luo H., Zhang Y., Li Q., Zhu Y., Xu J., Li Y., Song C., Wang B. (2016). Analysis of the Genome Sequence of the Medicinal Plant *Salvia miltiorrhiza*. Mol. Plant.

[27] Song Z., Lin C., Xing P., Fen Y., Jin H., Zhou C., Gu Y.Q., Wang J., Li X. (2020). A High-quality Reference Genome Sequence of *Salvia miltiorrhiza* Provides Insights into Tanshinone Synthesis in Its Red Rhizomes. Plant Genome.

[28] Wang Y., Guo B., Zhang F., Yao H., Miao Z., Tang K. (2007). Molecular Cloning and Functional Analysis of the Gene Encoding 3-Hydroxy-3-Methylglutaryl Coenzyme A Reductase from Hazel (*Corylus avellana* L. Gasaway). BMB Rep..

[29] Ha S.-H., Kim J.-B., Hwang Y.-S., Lee S.-W. (2003). Molecular Characterization of Three 3-Hydroxy-3-Methylglutaryl-CoA Reductase Genes Including Pathogen-Induced Hmg2 from Pepper (*Capsicum annuum*). Biochim. Biophys. Acta BBA Gene Struct. Expr..

[30] Shen G., Pang Y., Wu W., Liao Z., Zhao L., Sun X., Tang K. (2006). Cloning and Characterization of a Root-Specific Expressing Gene Encoding 3-Hydroxy-3-Methylglutaryl Coenzyme a Reductase from *Ginkgo biloba*. Mol. Biol. Rep..

[31] Li W., Liu W., Wei H., He Q., Chen J., Zhang B., Zhu S. (2014). Species-Specific Expansion and Molecular Evolution of the 3-Hydroxy-3-Methylglutaryl Coenzyme A Reductase (HMGR) Gene Family in Plants. PLoS ONE.

[32] Ma X., Luo N., Bai W., Wang X., Wang C., Cheng N., Liu H. (2023). Genome-Wide Analysis of the PtHMGR Gene Family and Functional Validation of PtHMGR5 Improving Drought Tolerance in *Populus trichocarpa*. Environ. Exp. Bot..

[33] Liu W., Zhang Z., Li W., Zhu W., Ren Z., Wang Z., Li L., Jia L., Zhu S., Ma Z. (2018). Genome-Wide Identification and Comparative Analysis of the 3-Hydroxy-3-Methylglutaryl Coenzyme A Reductase (HMGR) Gene Family in Gossypium. Molecules.

[34] Liu H., Zhou S., Chen P., Liu J., Huo K.-G., Han L. (2024). Exploring Genomic Large Language Models: Bridging the Gap between Natural Language and Gene Sequences. bioRxiv.

[35] Enjuto M., Balcells L., Campos N., Caelles C., Arró M., Boronat A. (1994). Arabidopsis Thaliana Contains Two Differentially Expressed 3-Hydroxy-3-Methylglutaryl-CoA Reductase Genes, Which Encode Microsomal Forms of the Enzyme. Proc. Natl. Acad. Sci. USA.

[36] Chen T., Yang M., Cui G., Tang J., Shen Y., Liu J., Yuan Y., Guo J., Huang L. (2024). IMP: Bridging the Gap for Medicinal Plant Genomics. Nucleic Acids Res..

[37] Pan W., Liu X., Li D., Zhang H. (2022). Establishment of an Efficient Genome Editing System in Lettuce Without Sacrificing Specificity. Front. Plant Sci..

[38] Wang Y., Shen Y., Shen Z., Zhao L., Ning D., Jiang C., Zhao R., Huang L. (2016). Comparative Proteomic Analysis of the Response to Silver Ions and Yeast Extract in *Salvia miltiorrhiza* Hairy Root Cultures. Plant Physiol. Biochem..

[39] Liu S., Yang S., Su P. (2024). Chemo-Enzymatic Synthesis of Bioactive Compounds from Traditional Chinese Medicine and Medicinal Plants. Sci. Tradit. Chin. Med..

[40] Zhang B., Li X., Li X., Lu Z., Cai X., Ou Yang Q., Ma P., Dong J. (2020). Lipopolysaccharide Enhances Tanshinone Biosynthesis via a Ca2+-Dependent Manner in *Salvia miltiorrhiza* Hairy Roots. Int. J. Mol. Sci..

[41] Fathi E., Majdi M., Dastan D., Maroufi A. (2019). The Spatio-Temporal Expression of Some Genes Involved in the Biosynthetic Pathways of Terpenes/Phenylpropanoids in Yarrow (*Achillea millefolium*). Plant Physiol. Biochem..

[42] Anwar M., Chen L., Xiao Y., Wu J., Zeng L., Li H., Wu Q., Hu Z. (2021). Recent Advanced Metabolic and Genetic Engineering of Phenylpropanoid Biosynthetic Pathways. Int. J. Mol. Sci..

[43] Ma P., Pei T., Lv B., Wang M., Dong J., Liang Z. (2022). Functional Pleiotropism, Diversity, and Redundancy of *Salvia miltiorrhiza* Bunge JAZ Family Proteins in Jasmonate-Induced Tanshinone and Phenolic Acid Biosynthesis. Hortic. Res..

[44] Wang M., Wang Y., Li X., Zhang Y., Chen X., Liu J., Qiua Y., Wang A. (2024). Integration of Metabolomics and Transcriptomics Reveals the Regulation Mechanism of the Phenylpropanoid Biosynthesis Pathway in Insect Resistance Traits in *Solanum habrochaites*. Hortic. Res..

[45] Zhou Z., Feng J., Huo J., Qiu S., Zhang P., Wang Y., Li Q., Li Y., Han C., Feng X. (2024). Versatile CYP98A Enzymes Catalyse *Meta* -hydroxylation Reveals Diversity of Salvianolic Acids Biosynthesis. Plant Biotechnol. J..

[46] Fu F., Qin H., Xin Y., Li Q., Kang H., Han L., Hua W., Cao X. (2025). Characterization of 4-Coumarate CoA Ligase (4CL) Gene Family and Functional Study of Sm4CL2/3/7/9 in *Salvia miltiorrhiza*. Funct. Integr. Genom..

[47] Jiang Y.Y., Tang Y.N., Tan Y.P., Sun S.F., Guo J., Cui G.H., Tang J.F. (2025). Construction of a multigene expression system for plants and verification of its function. China J. Chin. Mater. Med..

[48] Kumar S., Stecher G., Suleski M., Sanderford M., Sharma S., Tamura K. (2024). MEGA12: Molecular Evolutionary Genetic Analysis Version 12 for Adaptive and Green Computing. Mol. Biol. Evol..

[49] Cañizares E., Acién J.M., Gumuş B.Ö., Vives-Peris V., González-Guzmán M., Arbona V. (2024). Interplay between Secondary Metabolites and Plant Hormones in Silver Nitrate-Elicited Arabidopsis thaliana Plants. Plant Physiol. Biochem..

[50] Yang D., Fang Y., Xia P., Zhang X., Liang Z. (2018). Diverse Responses of Tanshinone Biosynthesis to Biotic and Abiotic Elicitors in Hairy Root Cultures of *Salvia miltiorrhiza* and *Salvia castanea* Diels f. *tomentosa*. Gene.

