# Supplementary material for: Divergent Roles of SmHMGR2 and a Novel SmHMGR5 in Tanshinone Biosynthesis Revealed by CRISPR/Cas9-Mediated Knockout in Salvia miltiorrhiza
Source: Int J Mol Sci. 2026 Apr 13;27(8):3485. doi: 10.3390/ijms27083485 (PMC13116991; doi:10.3390/ijms27083485)
Supplement: Supplementary file 1 [file ijms-27-03485-s001.zip › Supplementary Figure.pdf]

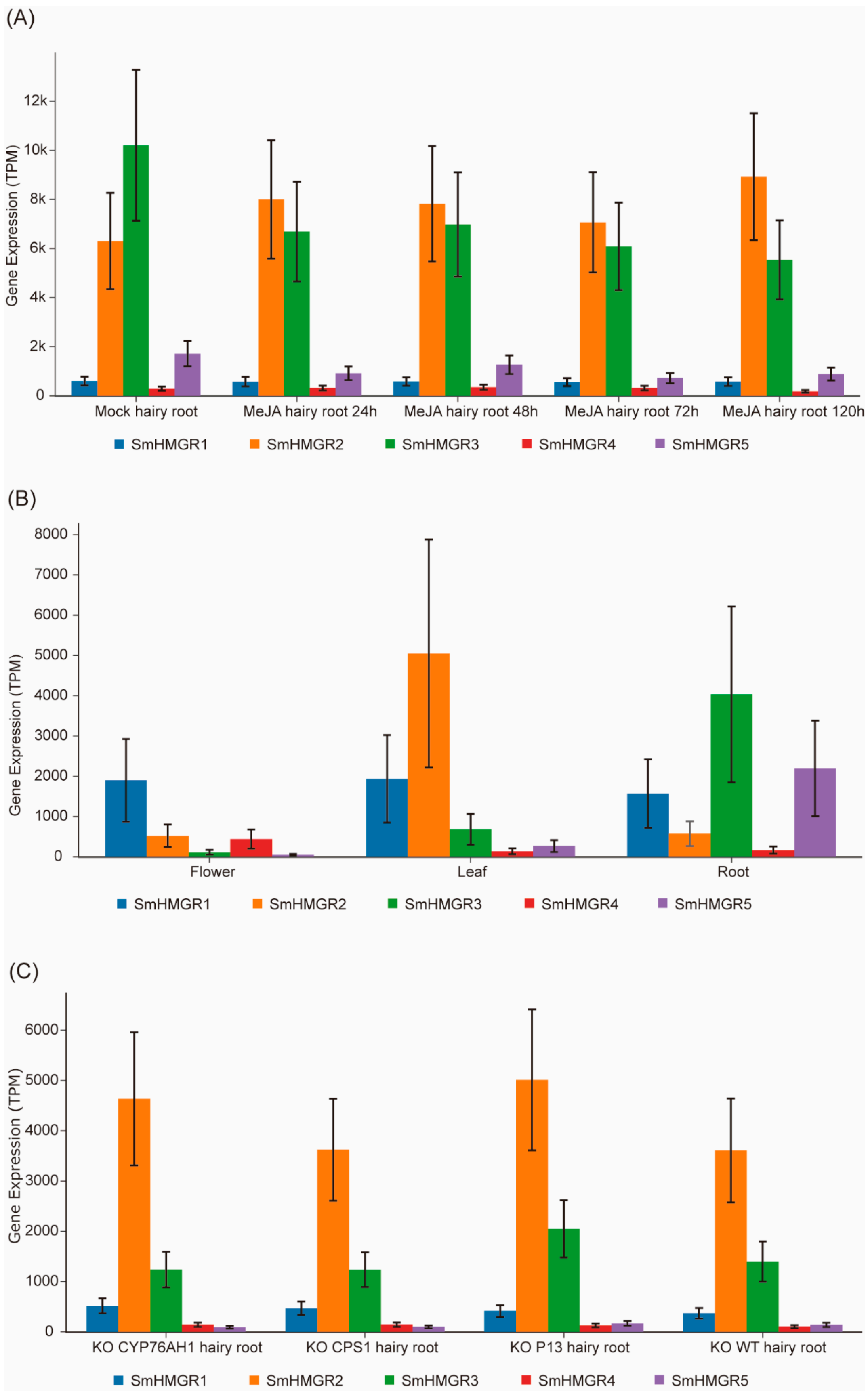


**Figure S1. Expression profiles of the five *SmHMGR* genes in different experimental systems.** (A) Expression levels of SmHMGR1-SmHMGR5 in mock-treated hairy roots and MeJA-treated

hairy roots at 24 h, 48 h, 72 h, and 120 h. (B) Tissue-specific expression of *SmHMGR1* – *SmHMGR5* in flower, leaf, and root tissues in an unknown purple-flower line. (C) Expression levels of *SmHMGR1-SmHMGR5* in CYP76AH1-knockout (KO), CPS1-KO, P13-KO, and wild-type (WT) hairy roots of bh2-7. All data are presented as mean ± standard deviation (SD) of three biological replicates, with gene expression quantified as transcripts per million (TPM).

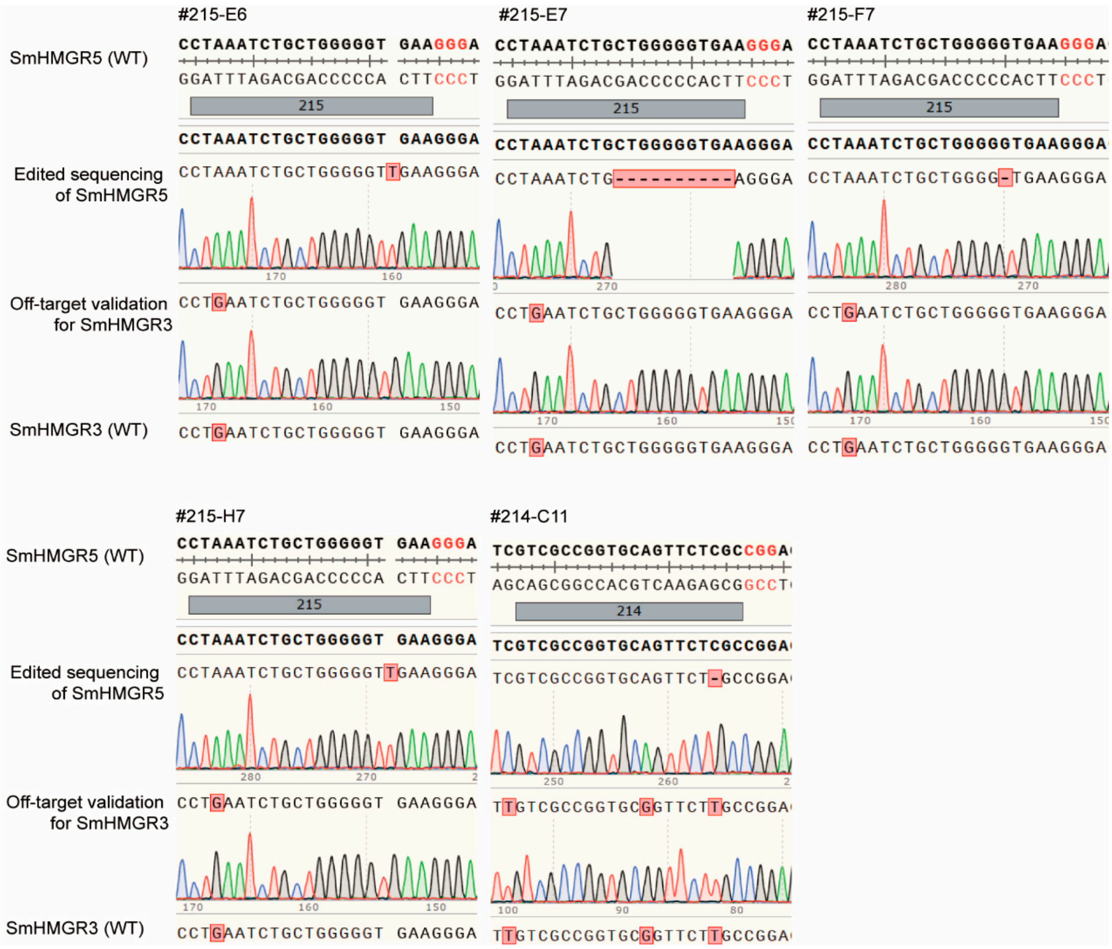


**Figure S2. Validation of off-target effects of sgRNA-214/215 on *SmHMGR3*.** The sequences are presented from top to bottom as follows: wild-type SmHMGR5 sequence; Sequencing results of *SmHMGR5* mutant lines 214-C11, 215-E6, 215-E7, 215-F7 and 215-H7; Sequences amplified from the corresponding mutant lines using SmHMGR3-specific primers; and wild-type *SmHMGR3* sequence.

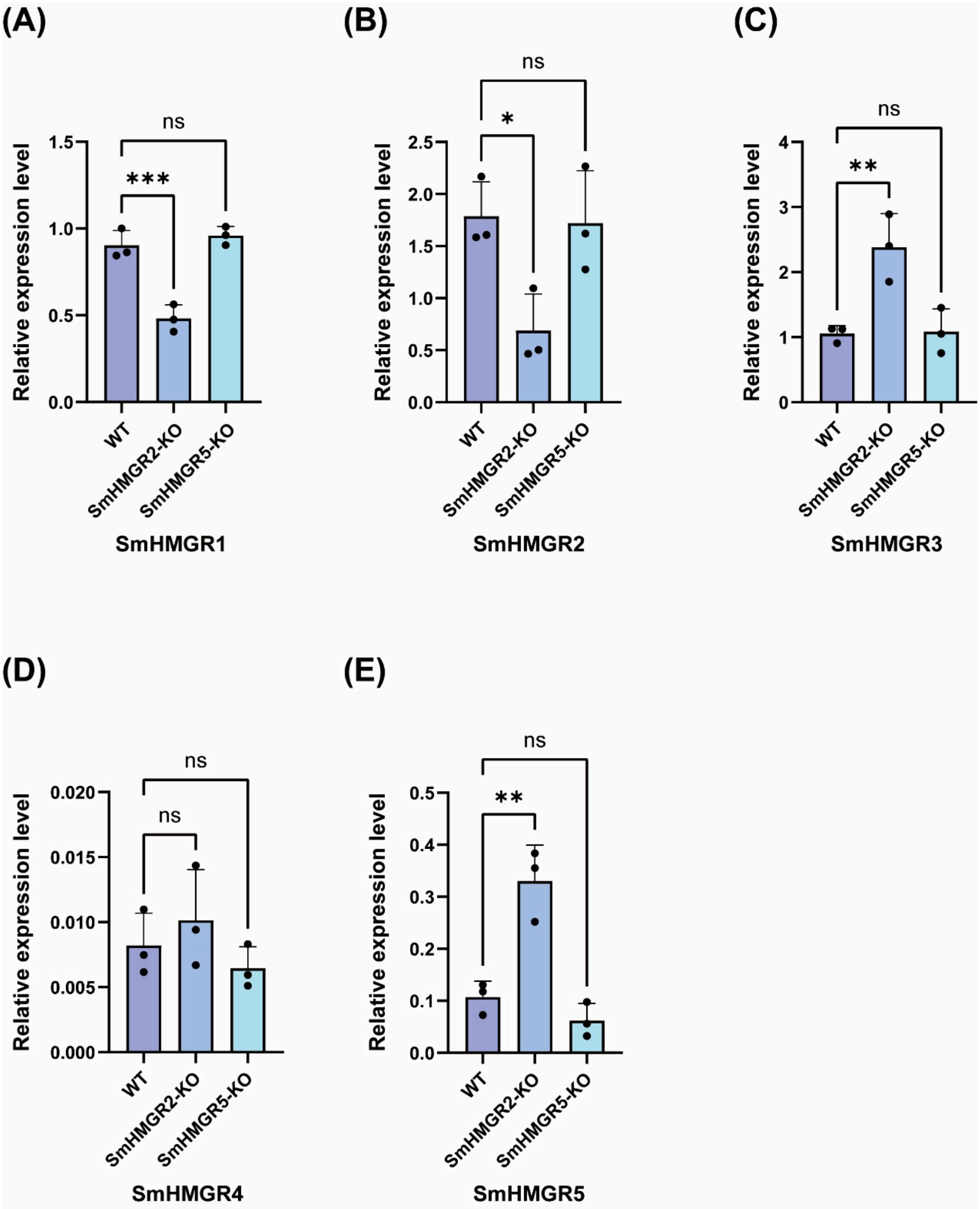


**Figure S3. Relative expression levels of *SmHMGR1–5* in *SmHMGR2*-KO and *SmHMGR5*-KO mutant lines.** (A–E) qRT-PCR analysis of *SmHMGR1* (A), *SmHMGR2* (B), *SmHMGR3* (C), *SmHMGR4* (D), and *SmHMGR5* (E) transcript levels in wild-type (WT) and knockout lines. Relative expression levels were calculated using the $2^{-\Delta\Delta Ct}$ method with *SmActin* as the internal reference. Significance was determined by one-way ANOVA followed by Tukey's post-hoc test: * $P < 0.05$, ** $P < 0.01$, *** $P < 0.001$; ns, not significant ($P > 0.05$).

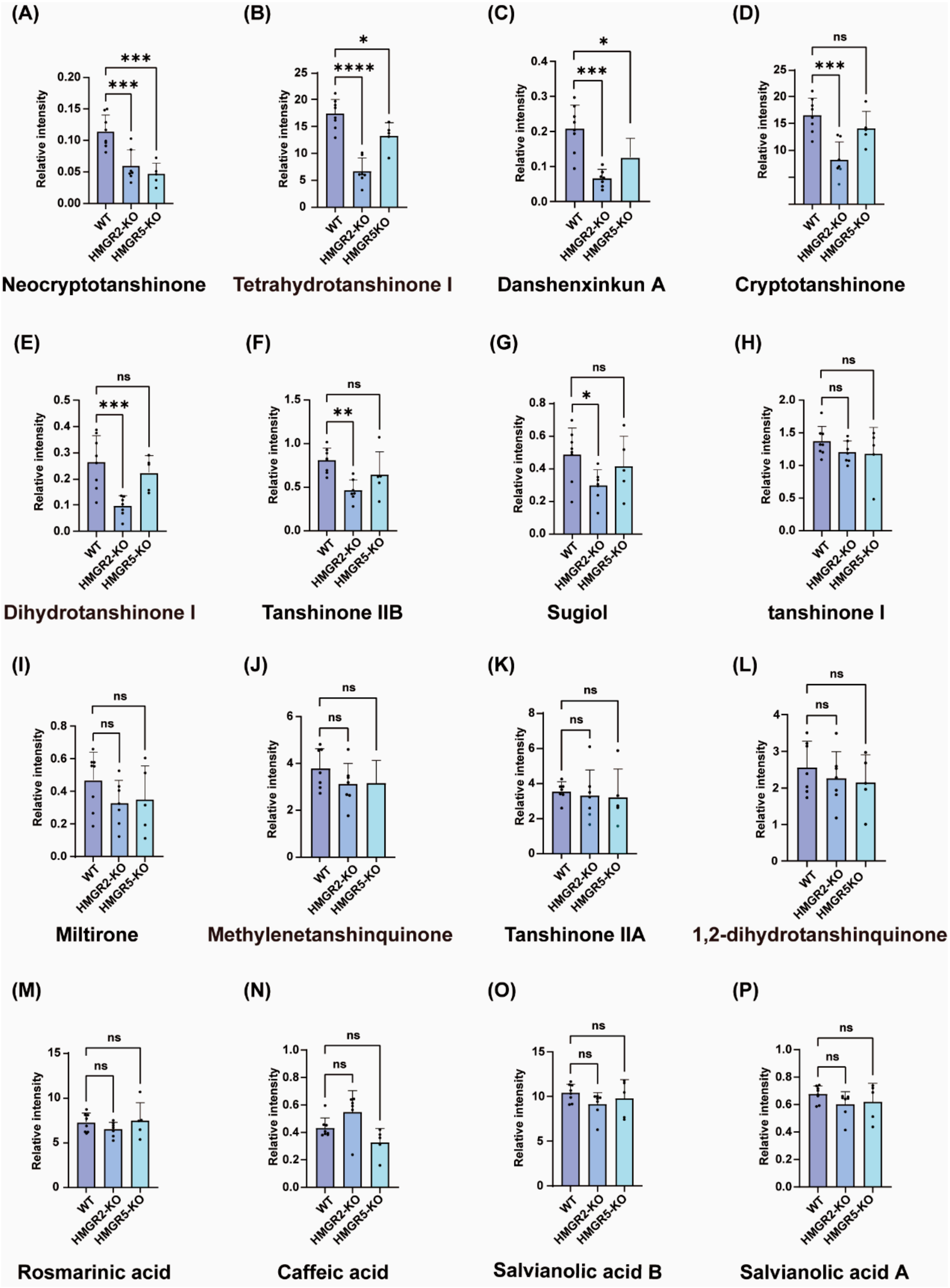


**Figure S4. Effects of *SmHMGR2* and *SmHMGR5* knockout on the accumulation of target metabolites in *Salvia miltiorrhiza* hairy roots.** Shown are the relative levels of 12 tanshinones and 4 phenolic acids in wild-type (WT), *SmHMGR2*-knockout (*SmHMGR2*-KO), and *SmHMGR5*-knockout (*SmHMGR5*-KO) hairy roots. Panels A-P represent the following metabolites: A, Miltirone; B, Tanshinone IIB; C, Danshenxinkun A; D, Dihydrotanshinone I; E, Tetrahydrotanshinone I; F, Neocryptotanshinone; G, Tanshinone I; H, Sugiol; I, 1,2-Dihydrotanshinquinone; J, Methylenetanshinquinone; K, Tanshinone IIA; L, Cryptotanshinone;

M, Caffeic acid; N, Rosmarinic acid; O, Salvianolic acid A; P, Salvianolic acid B. Significance was determined by one-way ANOVA followed by Tukey's post-hoc test: * $P < 0.05$, ** $P < 0.01$, *** $P < 0.001$, ****$p < 0.0001$; ns, not significant ($P > 0.05$).

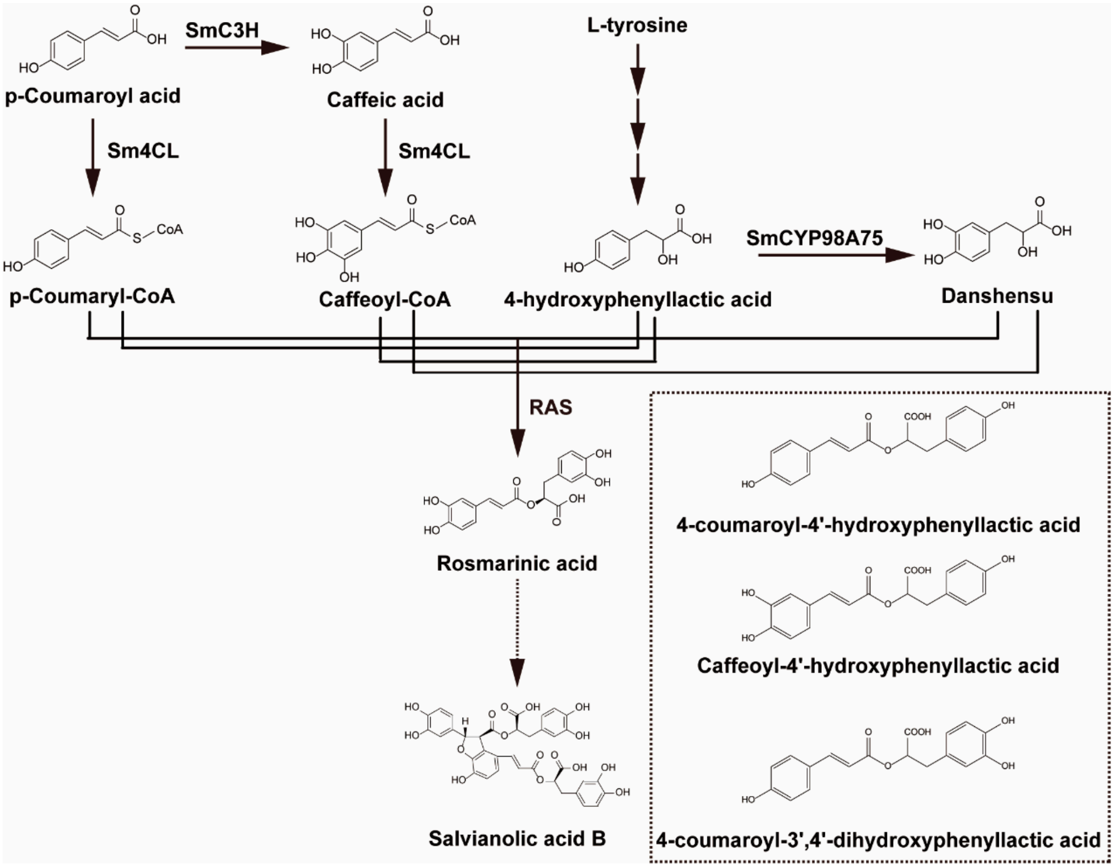


**Figure S5. Metabolic flux redirection and precursor competition regulation in the salvianolic acid biosynthetic pathway.** Solid arrows indicate confirmed biosynthetic steps, and multistage arrows indicate multi-step consecutive reactions. Compounds in dashed boxes represent intermediates formed by esterification between acyl donors (p-coumaroyl-CoA, caffeoyl-CoA) and acyl acceptors (danshensu, 4-hydroxyphenyllactic acid) for rosmarinic acid biosynthesis.
